# PRISM-G: an interpretable privacy scoring framework for assessing risk in synthetic human genome data

**DOI:** 10.1093/bioinformatics/btag377

**Published:** 2026-08-21

**Authors:** Alejandro Correa Rojo, Yves Moreau, Gökhan Ertaylan

**Affiliations:** ESAT-STADIUS, KU Leuven, Leuven, 3001, Belgium; ESAT-STADIUS, KU Leuven, Leuven, 3001, Belgium; Environmental Intelligence (EI), Flemish Institute for Technological Research (VITO), Mol, 2400, Belgium

## Abstract

**Motivation:**

Synthetic genomic data promises broader data access, but unresolved privacy risks remain a major concern. Existing evaluations often rely on similarity-based metrics that measure proximity between real and synthetic genomes, overlooking additional mechanisms through which genomic information may leak.

**Results:**

We introduce PRISM-G, a model-agnostic framework that quantifies privacy exposure in synthetic genomic data across three complementary components: proximity to real genomes in genetic-coordinate space, replay of familial or population-structure patterns, and trait-linked exposure through rare variants and membership-inference signals. These components are normalized and combined through a risk-averse aggregation into a single 0–100 PRISM-G score. By pairing PRISM-G with downstream utility metrics, the framework also enables analysis of privacy–utility trade-offs across generative models. We evaluated PRISM-G on synthetic cohorts generated by a generative adversarial network (GAN), a restricted Boltzmann machine (RBM), and a logic-based SAT solver (Genomator). Our results show that privacy vulnerabilities arise along different axes across models and marker densities, demonstrating that a single similarity-based metric is insufficient to characterize genomic privacy risk.

**Availability and implementation:**

The source code of PRISM-G is available at https://github.com/alejocrojo09/prismg.

## Introduction

The increasing availability of population-scale genomic resources has transformed biomedical research by enabling systematic characterization of human genetic variation and its role in health and disease (Venturini *et al.* 2025). Large international sequencing initiatives and national biobanks now host datasets comprising large collections of human genomes, supporting applications ranging from rare disease gene discovery to population-scale studies of complex traits and the development of precision medicine (Chen *et al.* 2025, Mani *et al.* 2025). However, the growing availability of genomic data also raises persistent concerns regarding privacy and responsible data sharing. Human genomes encode highly sensitive information and remain inherently identifiable even when explicit identifiers are removed, creating risks of re-identification or attribute disclosure (Conduah *et al.* 2025, Evans and Jarvik 2018, Shabani and Marelli 2019, Gymrek *et al.* 2013).

These concerns are particularly relevant in collaborative research environments where genomic data must be shared across institutions and national borders (Bonomi *et al.* 2020, Stark *et al.* 2025). In Europe, large-scale genomic research infrastructures operate across multiple jurisdictions, where differences in governance frameworks and national interpretations of data protection obligations complicate cross-border data sharing (Crum *et al.* 2026). Although initiatives such as the **European Health Data Space (EHDS)** (https://health.ec.europa.eu/ehealth-digital-health-and-care/european-health-data-space-regulation-ehds_en) and the **European Genomic Data Infrastructure (GDI)** (https://gdi.onemilliongenomes.eu/) aim to facilitate the secure secondary use of health and genomic data across member states, practical data sharing often remains constrained by disagreements over acceptable privacy risks and by the lack of widely accepted technical evidence demonstrating that shared genomic data cannot be linked back to individuals (Royo *et al.* 2025).

In response to these challenges, synthetic data generation has emerged as a strategy to expand data accessibility while reducing privacy risks and preserving analytical utility without direct reliance on real individual-level records (Giuffrè and Shung 2023). In genetics, several generative approaches, including haplotype resampling (Wharrie *et al.* 2023), deep learning architectures (Yelmen *et al.* 2021, 2023, Kenneweg *et al.* 2025), and logic-based methods (Burgess *et al.* 2025), have been developed to produce artificial genotype datasets that reproduce key genomic properties such as allele frequencies, linkage disequilibrium (LD), and population structure. These synthetic datasets can support exploratory analyses, method development, and collaborative research where access to individual genomic data is restricted, while enabling applications in rare disease research, population genetics, and genetic risk estimation (Mendes *et al.* 2025).

Despite these advances, evaluating the privacy risk of synthetic genomic datasets remains challenging. Many studies rely on distance-based similarity measurements to estimate how closely synthetic genomes resemble individuals in the original dataset. Typical approaches include nearest-neighbor or Hamming distance comparisons between real and synthetic genomes, and low similarity is often interpreted as evidence that synthetic datasets are privacy-preserving (Yelmen *et al.* 2021, 2023, Wharrie *et al.* 2023, Burgess *et al.* 2025, Kenneweg *et al.* 2025). However, this assumption can be misleading. Previous studies have shown that Membership Inference Attacks (MIAs) can determine whether a specific individual was used to train a generative model even when synthetic genomes exhibit low similarity to the training data (Oprisanu *et al.* 2022).

These limitations arise because genomic information can reveal identity through mechanisms beyond simple genome similarity. Synthetic datasets may reproduce relatedness patterns from the training data, enabling familial or genealogical matching (Edge and Coop, 2020). Distinctive features, such as rare variants or unusual mutation combinations, may uniquely characterize individuals or small related groups (Thomas *et al.* 2024). Personal information may also be inferred through linkage with external resources, including functional genomics data (Gürsoy *et al.* 2022). As a result, datasets that appear safe under similarity-based evaluations may still retain signals enabling indirect identification. These observations suggest that privacy exposure cannot be captured by a single metric; rather, risk arises across complementary representations of genetic information, such as population structure, relatedness, and distinctive variant patterns, requiring evaluation frameworks that assess these signals jointly.

Building on this perspective, we introduce **PRISM-G (Privacy Risk Integrated Score for Multi-representation Genomes)**, a framework for systematically assessing privacy exposure in synthetic genomic datasets. PRISM-G evaluates privacy risk across complementary genomic representations that capture proximity between real and synthetic genomes, patterns of genetic relatedness, and trait-linked genetic features, producing a privacy score that enables consistent comparison across synthetic datasets and generative models. Beyond providing a compact privacy metric, the framework generates interpretable diagnostics that reveal the mechanisms underlying potential privacy leakage.

To illustrate this approach, we applied PRISM-G to synthetic cohorts generated from multiple generative model families, including generative adversarial networks (GANs), restricted Boltzmann machines (RBMs), and a logic-based SAT solver, using data subsets from the 1000 Genomes Project (1000 Genomes Project Consortium 2015).

## PRISM-G framework: algorithmic background and overview

Genomic data can be represented through complementary statistical structures that capture population variation and relatedness. Common representations include coordinate embeddings derived from dimensionality reduction, relational representations based on genetic relationship matrices (GRMs), and feature-level signals such as rare genetic variants associated with individual characteristics (Patterson *et al.* 2006, Madsen and Browning 2009, Goudet *et al.* 2018). These representations are widely used in population genetics to quantify genetic similarity, infer relatedness, and characterize variant distributions across individuals.

Building on these genomic representations, we developed PRISM-G, which evaluates privacy exposure by comparing real genomes with synthetic cohorts across three complementary components: the **Proximity Leakage Index (PLI)**, measuring similarity between real and synthetic genomes; the **Kinship Replay Index (KRI)**, capturing replication of relatedness patterns such as kinship and haplotype sharing; and the **Trait-Linked Leakage Index (TLI)**, detecting preservation of rare-variant signals. Each component reflects a distinct representation of genomic variation and is decomposed into interpretable submetrics that diagnose specific leakage mechanisms. A structured comparison of the algorithmic foundations of these components and their relation to existing genomic and privacy metrics is provided in the Supplementary Data.

**Figure 1:**
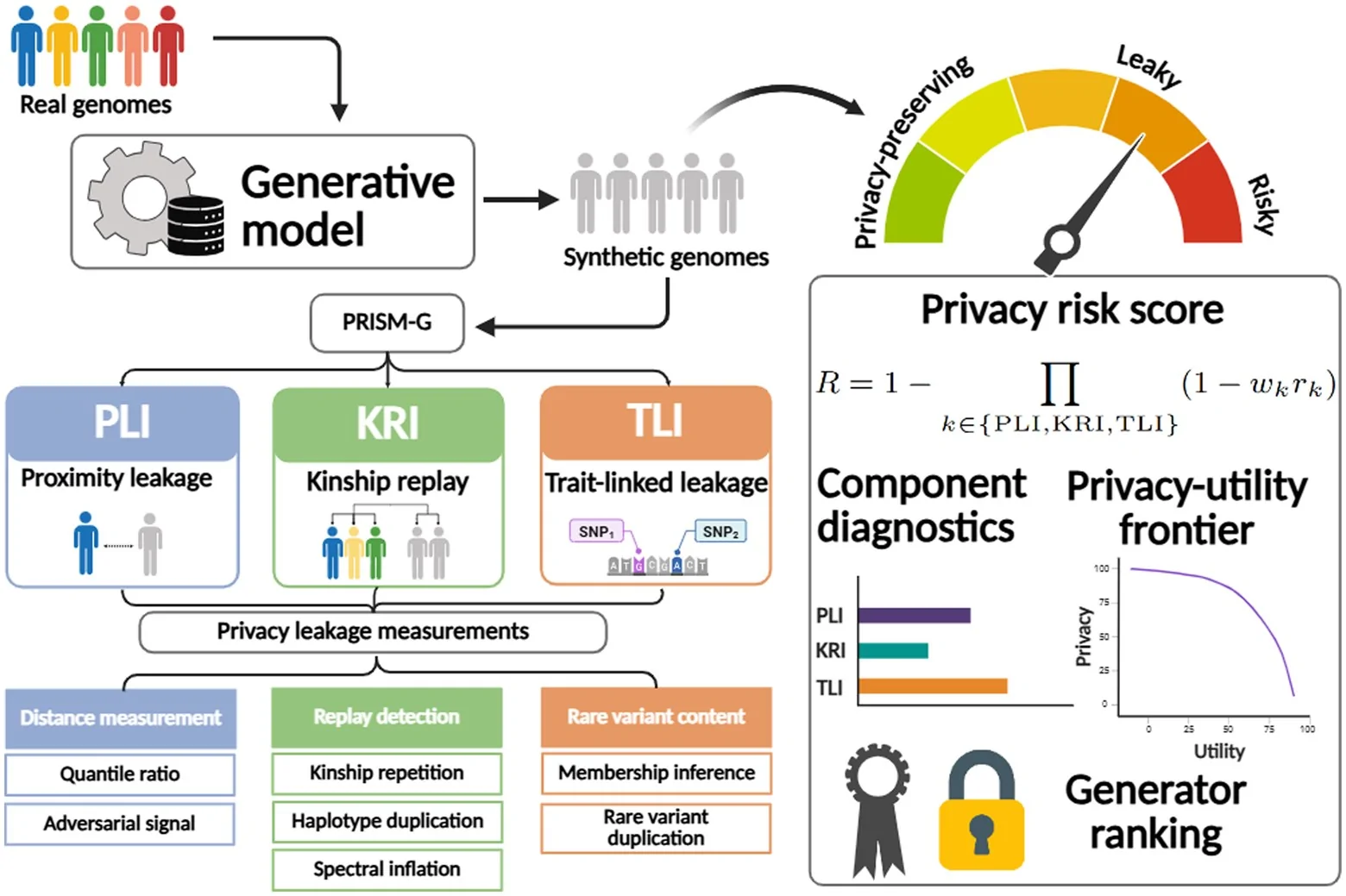
Overview of the PRISM-G framework. Imaged generated with BioRender.

The component metrics are subsequently aggregated into a single composite score summarizing the overall privacy risk of a synthetic dataset. This unified score enables statistical comparison of datasets generated by different models and provides a quantitative measure of privacy exposure. When combined with downstream utility measurements, the PRISM-G score can also be used to construct privacy–utility frontiers that characterize trade-offs between analytical utility and privacy risk. An overview of the PRISM-G workflow is illustrated in Fig. 1.

In the following sections we formally define each component metric and the aggregation procedure used to derive the PRISM-G score, while the full mathematical formulation and methodological details are provided in the Supplementary Data.

### Component metrics

#### Proximity Leakage Index

The PLI metric evaluates whether synthetic genomes lie unusually close to real individuals in genetic coordinate space. Genotypes are first standardized by allele frequencies and embedded using principal component analysis (PCA), producing a low-dimensional representation of population structure (Patterson *et al.* 2006). Distances between real and synthetic samples are then computed using nearest-neighbor Euclidean distance in the principal component space. From these distances, PLI combines two complementary submetrics:

- **Quantile proximity ratio (** $r_{p}$ **):** evaluates whether the smallest distances between synthetic and real individuals fall below the expected distribution of distances among real individuals.
- **Adversarial proximity (** $r_{A}$ **):** measures whether holdout individuals are closer to synthetic genomes than to other real individuals.

Together, these diagnostics detect identity proximity, where synthetic samples may be sufficiently similar to real genomes to enable singling-out or linkage with external genetic databases. The overall score is defined as


(1) $$\text{PLI}=\max(r_{p},r_{A}).$$

#### Kinship Replay Index

The KRI metric captures privacy risks arising from the reproduction of relational genetic structure. Genotypes are used to estimate the GRM following the method proposed by VanRaden, which quantifies pairwise relatedness among individuals (VanRaden 2008). In addition, local haplotype patterns derived from genotype windows are used to identify repeated genotype segments indicative of shared ancestry (Oldoni *et al.* 2019). It aggregates four diagnostics derived from the GRM and haplotype structure:

- **Replay (** $r_{\text{replay}}$ **):** evaluates whether the distribution of close-kin relationships observed in the real dataset is reproduced in the synthetic cohort.
- **Internal kinship excess (** $r_{\text{IKE}}$ **):** measures whether synthetic samples exhibit elevated mutual relatedness relative to the real data by comparing the fraction of kinship values exceeding predefined thresholds.
- **Micro-haplotype collisions (** $r_{\text{HAP}}$ **):** detects repeated short genotype patterns by scanning sliding windows of SNPs and quantifying identical haplotype segments shared across synthetic individuals.
- **Spectral inflation (** $r_{\text{SPEC}}$ **):** identifies concentration of relatedness structure through inflation of the leading eigenvalue of the GRM.

Together these diagnostics capture structural replay, where generative models reproduce genealogical patterns that may enable familial matching or inference of hidden relationships. The overall score is defined as


(2) $$\text{KRI}=\max(r_{\text{replay}},r_{\text{IKE}},r_{\text{HAP}},r_{\text{SPEC}}).$$

#### Trait-Linked Leakage Index

The TLI metric measures privacy exposure arising from distinctive feature-level signals encoded in rare variants. Rare variants can act as highly identifying genomic features and may reveal sensitive information when preserved in synthetic datasets. TLI therefore evaluates whether rare variant burden patterns and uncommon variant signals observed in real genomes are reproduced in synthetic samples (Madsen and Browning 2009). It combines two signals:

- **Membership inference signal (** $r_{\text{mia}}$ **):** evaluates whether individuals used to train the generative model can be distinguished from holdout individuals using rare variant burden scores and a nearest-neighbor membership classifier.
- **Rare variant uniqueness (** $r_{\text{uniq}}$ **):** detects whether uncommon variants appear more frequently in the synthetic cohort than expected.

Together these signals summarize the extent to which rare variant patterns are preserved in the synthetic dataset. The overall score is defined as


(3) $$\text{TLI}=\max(r_{\text{mia}},r_{\text{uniq}}).$$

### Aggregation of privacy risk

Each component produces a normalized risk score in the interval [0,1]. Because the three components represent distinct leakage mechanisms, PRISM-G aggregates them using a risk-averse noisy-OR formulation:


(4) $$R_{\text{raw}}=1-\prod_{k\in \{\text{PLI},\text{KRI},\text{TLI}\}}\left(1-w_{k}r_{k}\right)$$

where $r_{k}$ denotes the component risk and $w_{k}$ are non-negative weights constrained to sum to one.

The noisy-OR formulation reflects that privacy exposure may arise through multiple alternative leakage pathways, including proximity, kinship structure, or rare-variant leakage. This differs from the within-component max operator, which conservatively flags any strong submetric-level signal. When average-case rather than worst-case exposure is preferred, PRISM-G can alternatively use mean or median submetric aggregation. Future extensions may also explore parameterized power-mean aggregation to interpolate between average-case and worst-case risk summaries.

Raw privacy scores depend on dataset characteristics such as cohort size, allele-frequency spectrum, and population structure, and therefore cannot be directly compared across experiments. PRISM-G therefore calibrates raw risk values using reference generators that define empirical lower and upper bounds of privacy exposure.

Two anchors are used: a *safe baseline generator*, which preserves allele frequencies while removing correlation structure, and a *leaky reference generator*, which intentionally replicates training samples and reproduce their relational structure. These anchors define calibration parameters α and β, representing the expected raw risk under the safe and leaky references, respectively. Raw risk values are then mapped onto a standardized 0–100 PRISM-G privacy scale:


(5) $$\text{PRISM}-G=100\times \frac{R_{\text{raw}}-\alpha }{\beta -\alpha }$$

To compare privacy risk across generators, PRISM-G evaluates generator rankings using Kendall’s rank correlation coefficient. Rankings are recomputed across bootstrap samples and compared with the baseline ranking obtained from the full dataset. The average Kendall correlation measures the agreement between rankings and reflects the stability of the resulting PRISM-G ordering.

The component weights $w_{k}$ are calibrated using ridge regularization based on the anchor generators. Hyperparameters are explored via grid search, and candidate configurations are evaluated by the Kendall ranking stability. The configuration that maximizes the average Kendall correlation is selected to produce the final PRISM-G scores. The resulting score can be stratified into predefined risk categories (e.g. quartiles) to facilitate interpretation and classify datasets as privacy-preserving, intermediate (leaky), or high-risk.

Because PRISM-G is calibrated using dataset-specific anchors, **absolute scores should not be interpreted as universal privacy probabilities.** Scores are most directly comparable across generators evaluated on the same dataset under the same calibration setting. Comparisons across datasets or cohorts should therefore recalibrate the anchors or report component-level PLI, KRI, and TLI values alongside the calibrated score.

### Privacy-utility analysis

The calibrated PRISM-G score provides a compact summary of privacy exposure, while component diagnostics reveal the mechanisms underlying potential leakage. Because generative models often trade fidelity for privacy protection, PRISM-G can also be paired with task-specific utility metrics to analyze privacy–utility trade-offs, enabling systematic comparison between models and identification of generators that achieve favorable balances between analytical utility and privacy protection.

## Evaluation

### Experimental setup

We evaluated PRISM-G on synthetic genomic cohorts generated by three model families: generative adversarial networks (GAN), restricted Boltzmann machines (RBM), and Genomator, a SAT-based genome generator (Burgess *et al.* 2025). GAN and RBM datasets were obtained from Yelmen *et al.* (2021), while Genomator cohorts were generated with varying Hamming distance constraints, which control the minimum number of variant differences allowed between synthetic and training samples.

All experiments used the 1000 Genomes Project Phase 3 (1KGP) dataset comprising 2504 individuals. Analyses were conducted on two genotype panels: a 10 000-SNP subset from Chromosome 15 and a 65 535-SNP subset from Chromosome 1. For each experiment, the real dataset was partitioned into training (80%) and holdout (20%) cohorts, and synthetic datasets were generated to match the size of the holdout set.

For calibration of PRISM-G scores, a synthetic dataset generated using a binomial sampler based on 1KGP allele frequencies served as the safe dataset, and a copycat dataset served as the leaky dataset. Component weights were calibrated via ridge regularization against these anchors using a grid search over calibration hyperparameters (Supplementary Data), and ranking stability was evaluated for each configuration using Kendall’s rank correlation across bootstrap replicates. This procedure also served as a sensitivity analysis, assessing how hyperparameter choices affect the dispersion and relative ordering of PRISM-G scores across generators.

To characterize population structure, PCA was applied to the real training data, generating embeddings that visualize genetic clusters using 1KGP population labels. Synthetic samples were projected onto the same coordinate space to ensure comparability between real and synthetic genomes. Population-structure preservation was quantified using Procrustes similarity between centroids of real and synthetic ancestry clusters in PCA space and Jensen–Shannon (JS) similarity derived from ancestry label distributions. Downstream predictive performance was evaluated using a train-real, test-synthetic (TRTS) protocol, where a random forest classifier trained on real genomes predicted ancestry labels in the synthetic datasets. We averaged Procrustes similarity and JS similarity as an overall utility metric capturing both structure preservation and predictive performance.

We present the main results for the Chromosome 1 subset (65 535 SNPs) and compare them with findings from the Chromosome 15 dataset (10 000 SNPs). A full description of the Chromosome 15 results is provided in the Supplementary Data.

### Privacy leakage diagnostics

**Figure 2:**
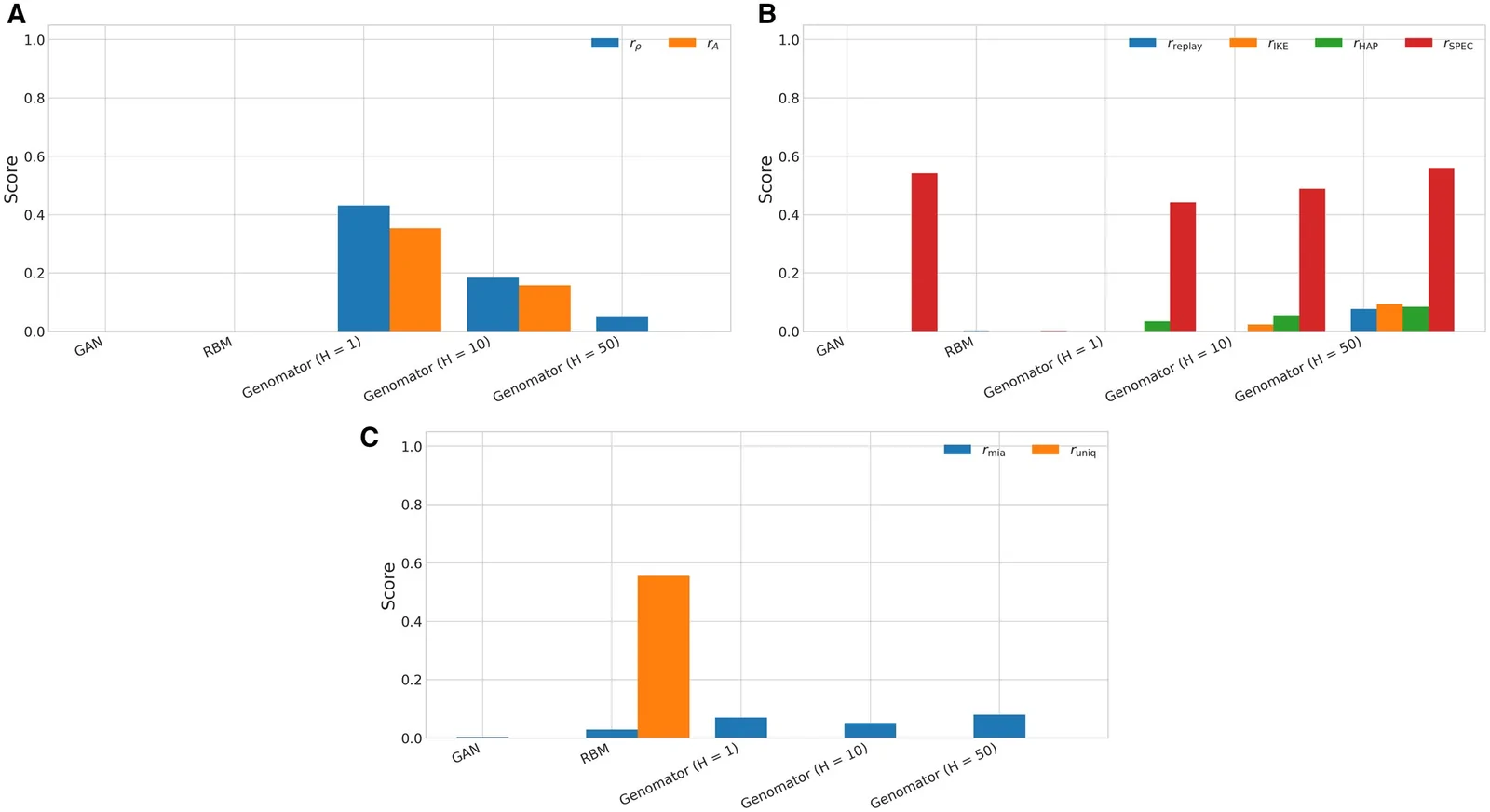
PRISM-G submetrics for Chromosome 1 dataset. Each barplot shows (A) Proximity leakage components. (B) Kinship replay components. (C) Trait leakage components.

We first evaluated PRISM-G submetrics for each synthetic dataset to illustrate how different generators exhibit distinct privacy leakage mechanisms prior to aggregation into PRISM-G scores. Figure 2 summarizes the privacy leakage submetrics for the Chromosome 1 dataset. On this dataset, PLI diagnostics indicated no proximity-based leakage for GAN and RBM (Fig. 2A). In contrast, Genomator showed a proximity signal that depended on the Hamming distance constraint H∈{1,10,50}. Under tight constraints (H=1), synthetic genomes appeared unusually close to holdout individuals, producing the strongest proximity signal. Increasing the constraint progressively reduced this effect, with proximity exposure decreasing at H=10 and becoming minimal at H=50.

Kinship replay diagnostics revealed generator-specific patterns (Fig. 2B). GAN showed moderate spectral inflation ($r_{\text{SPEC}}=0.541$), suggesting partial reproduction of global relatedness structure. In contrast, RBM exhibited negligible kinship leakage across all KRI submetrics. Genomator displayed a constraint-dependent pattern modulated by the Hamming distance parameter *H*: kinship-related signals increased as the constraint was relaxed, with higher *H* values showing stronger haplotype-collision and spectral signals ($r_{\text{HAP}}$ and $r_{\text{SPEC}}$), while replay signals remained comparatively modest. In the present experiments, $r_{\text{HAP}}$ should be interpreted as a local haplotype diagnostic over the assayed SNP panel, rather than a whole-genome identity-by-descent (IBD) estimate. For full-scale deployment, $r_{\text{HAP}}$ should be extended to genome-wide segments, as haplotype sharing depends on both marker density and genomic span.

For the TLI component, membership inference signals were generally low across models (Fig. 2C), although small $r_{\text{mia}}$ signals were observed for RBM and Genomator. Rare-variant uniqueness showed stronger differences: GAN and Genomator exhibited negligible $r_{\text{uniq}}$, whereas RBM displayed elevated rare-variant uniqueness ($r_{\text{uniq}}=0.555$), indicating stronger preservation of distinctive variant configurations.

Applying the same analysis to the Chromosome 15 dataset yielded similar qualitative patterns (Supplementary Fig. S1), although submetric values were generally higher for this lower-density SNP panel. Genomator again showed decreasing proximity signals with increasing *H*, kinship metrics were dominated by spectral-relatedness signals for RBM and Genomator, and RBM and GAN exhibited elevated rare-variant uniqueness. Detailed results for both datasets are provided in Supplementary Tables 1–6 in the Supplementary Data.

### Component-level PRISM-G scores

**Figure 3:**
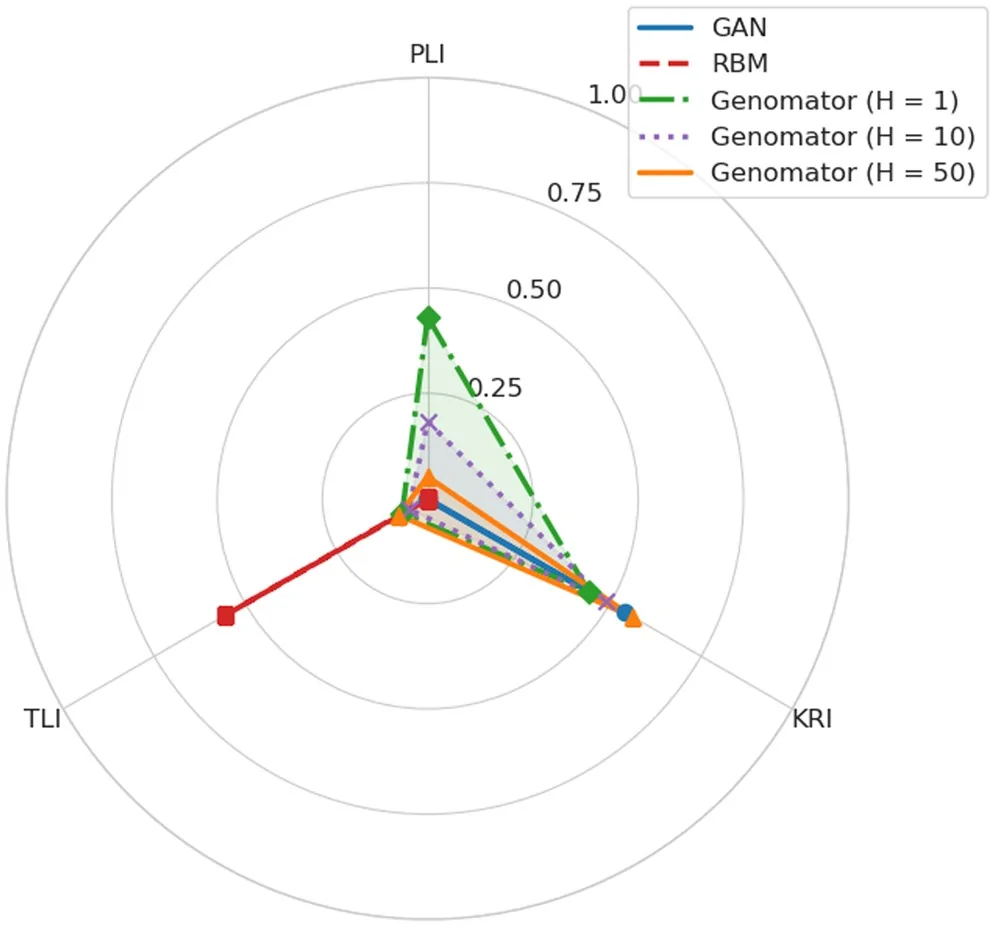
PRISM-G components (PLI, KRI, and TLI) for the Chromosome 1 dataset.

We next examined component-level PRISM-G scores to assess how proximity, relatedness, and trait-linked leakage contribute to overall privacy risk across generators. Figure 3 shows a radar plot of the component scores for the Chromosome 1 dataset. GAN and RBM showed negligible proximity leakage. GAN showed moderate relatedness (KRI=0.541), whereas RBM displayed low kinship replay (KRI=0.002). Trait-linked leakage remained minimal for GAN (TLI=0.003) but was elevated for RBM (TLI=0.555). Genomator showed a constraint-dependent profile, with proximity leakage decreasing as the Hamming constraint increased (0.052<PLI<0.431), intermediate kinship signals (0.441<KRI<0.560), and low trait-linked leakage (0.052<TLI<0.079).

Results for the Chromosome 15 dataset showed similar qualitative patterns (Supplementary Fig. S2): RBM exhibited the strongest trait-linked leakage, Genomator configurations were mainly driven by proximity and kinship signals varying with the *H* constraint, and GAN showed comparatively balanced component scores. Full estimates are provided in Supplementary Tables 7–9.

### PRISM-G score and privacy ranking

We first estimated PRISM-G scores using the reference anchors with default hyperparameters and performed a sensitivity analysis through grid search to assess the effect of calibration parameters on the estimated privacy risk (Supplementary Data). Under the default configuration, PRISM-G scores for the Chromosome 1 dataset yielded the following ranking from safer to riskier: **GAN > Genomator (**H=50**) > Genomator (**H=10**) > Genomator (**H=1**) > RBM**. Across the sensitivity analysis, ridge regularization primarily affected the dispersion of scores, particularly at higher marker densities, but did not alter the relative ordering of generators. Overall, rankings and qualitative conclusions remained consistent across the explored parameter grid, indicating that PRISM-G estimates are robust to plausible variations in calibration and regularization parameters (Supplementary Fig. S3). Complete estimates from the sensitivity analysis and ranking results are reported in Supplementary Tables 10–17.

**Figure 4:**
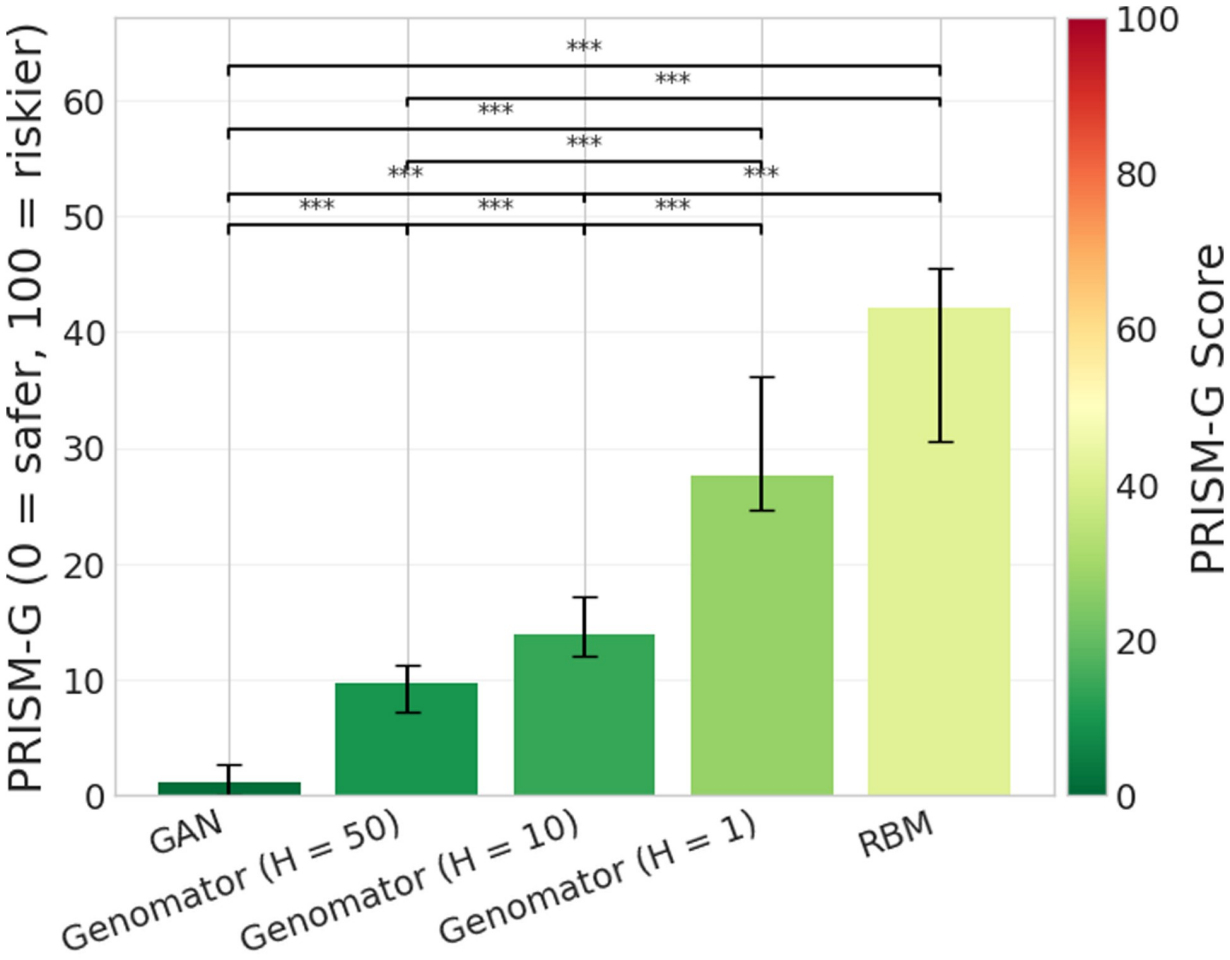
Mean PRISM-G scores for the Chromosome 1 dataset and pairwise comparisons obtained using grid search evaluation.

Using the hyperparameter configuration that maximized the average Kendall rank correlation across bootstrap replicates, we obtained the final PRISM-G estimates summarized in Figure 4 for the Chromosome 1 dataset. Scores ranged from 1.204 (GAN) to 42.034 (RBM), with Genomator configurations showing intermediate values that decreased as the Hamming distance constraint was relaxed (27.535 for H=1 to 9.628 for H=50). Stratifying the scores by quartiles of the PRISM-G scale, all datasets remained within the privacy-preserving range (PRISM-G<50). Rankings remained stable across the parameter grid (τ=0.985, P-value<0.018), consistently identifying GAN as the safest generator and RBM as the most privacy-leaky method. Similar patterns were observed for the Chromosome 15 dataset, although overall risk scores were higher, shifting RBM closer to the leaky reference (Supplementary Fig. S4).

### Privacy-utility trade off

**Figure 5:**
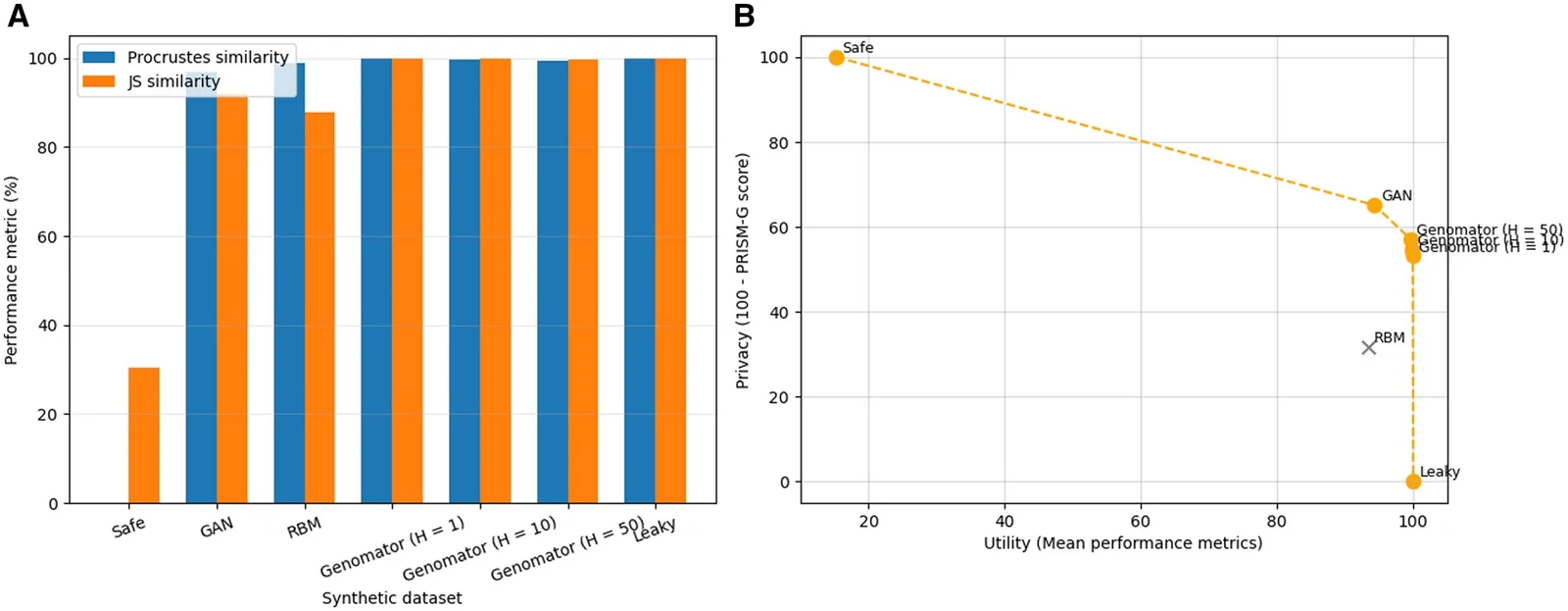
Performance metrics and privacy–utility trade-off for the Chromosome 1 dataset: (A) population structure preservation and ancestry inference utility; (B) privacy–utility frontier based on PRISM-G scores.

Using the estimated PRISM-G scores as privacy metrics, we constructed a privacy–utility frontier to evaluate downstream analytical performance while controlling privacy risk. As a representative task, we assessed genetic ancestry prediction using the procedure described in the Experimental Setup section. Figure 5 shows the obtained performance metrics and privacy-utility frontier for the Chromosome 1 dataset. Across generators, synthetic datasets preserved population structure and achieved high utility for ancestry prediction (Fig. 5A and Supplementary Figs. 5-6).

Pairing the PRISM-G scores with the average performance metrics as a utility measure yields the privacy–utility frontier shown in Fig. 5B. The Pareto front represents the set of non-dominated solutions, where improving privacy would require sacrificing utility and vice versa. Genomator configurations lie on this frontier, maintaining high utility while reducing privacy risk as the Hamming constraint increases. GAN also achieves high utility with moderate privacy risk. In contrast, RBM is dominated, exhibiting higher privacy risk without a corresponding utility advantage. Similar privacy–utility patterns were observed for the Chromosome 15 dataset (Supplementary Figs. 7-8).

## Discussion

Current evaluations of synthetic genomic data often rely on similarity-based diagnostics to argue that generated genomes differ sufficiently from real samples (Giuffrè and Shung 2023, Wharrie *et al.* 2023, Kenneweg *et al.* 2025, Royo *et al.* 2025). However, similarity alone does not capture risks such as singling-out, relatedness replay, or rare-variant leakage (Kaabachi *et al.* 2025). To address this gap, we introduced PRISM-G, which evaluates privacy risk across proximity, kinship, and trait-linked signals. Applying PRISM-G across multiple generators and SNP panels from the 1KGP dataset revealed distinct privacy profiles: GAN showed the lowest overall risk, RBM exhibited elevated rare-variant leakage, and Genomator displayed parameter-dependent trade-offs between proximity and relatedness signals. Overall, these findings highlight that privacy exposure arises through multiple mechanisms rather than a single similarity signal, supporting the need for domain-aware privacy evaluation frameworks (Pilgram *et al.* 2025, Zamzmi *et al.* 2025).

Beyond component-level diagnostics, PRISM-G also enables privacy–utility evaluation. Although all generators achieved similarly high utility for ancestry prediction, their privacy risks differed substantially. Genomator maintained high utility while reducing privacy risk as the Hamming distance constraint increased, whereas RBM showed higher privacy exposure without improved analytical performance. These results highlight that generators with similar utility may differ markedly in privacy risk, supporting the use of PRISM-G for model selection and parameter tuning.

Three practical insights emerge from our analysis. First, component-level diagnostics are essential, as similar aggregate scores may arise from different leakage mechanisms. Second, calibration against reference generators provides an interpretable privacy scale that facilitates comparison across models. Third, our results reinforce that privacy risk depends on dataset characteristics and generator settings, indicating that no single model is uniformly safest across contexts.

PRISM-G has several key limitations that constrain interpretation of its results. First, it is an empirical framework without formal privacy guarantees, and its outputs depend on the dataset, selected features, and calibration anchors; thus, **a low score reflects lower relative risk under the chosen setup rather than absence of privacy risk or evidence of non-memorization.** Second, it does not incorporate a demographic null model, so signals such as proximity, haplotype sharing, or IBD may reflect population structure (e.g. admixture, bottlenecks, or shared ancestry) rather than memorization. Third, its aggregation assumes independent leakage pathways, while these signals may be correlated; more flexible approaches such as regression, hierarchical, or copula-based models could better capture such dependencies. Finally, evaluations are limited to subsets of the 1KGP panel, limiting robustness assessment across populations; broader analyses across diverse cohorts and subgroups are needed to characterize generalizability and estimate null and expected metric distributions.

Future work should address these gaps by incorporating population-specific forward or coalescent simulations to better model expected metric distributions under realistic demographic histories, and by extending the framework with additional genomic features, alternative embeddings, richer trait-linked representations (including combinations of common variants), and expanded downstream applications such as genome-wide association studies and polygenic risk score modeling.

## Conclusions

Overall, PRISM-G provides a compact and interpretable approach for evaluating privacy risk in synthetic genomic datasets. By combining component-level diagnostics, calibrated risk scores, and privacy–utility analysis, the framework enables systematic comparison of synthetic genome generators towards informed decisions about the safe use and release of synthetic genomic data.

## Supplementary Material

btag377_Supplementary_Data

## Data Availability

The 1000 Genomes Project is an open-access dataset available at https://ftp.1000genomes.ebi.ac.uk/vol1/ftp/. All synthetic genomes used in this study have been deposited in the PRISM-G repository on GitHub at https://github.com/alejocrojo09/prismg.
