# Supplementary material for: PRISM-G: an interpretable privacy scoring framework for assessing risk in synthetic human genome data
Source: Bioinformatics. 2026 Aug 21;42(Suppl 2):btag377. doi: 10.1093/bioinformatics/btag377 (PMC13501313; doi:10.1093/bioinformatics/btag377)
Supplement: Supplementary file 1 [file btag377_supplementary_data.pdf]

Part I
Supplementary Methods

1 Algorithm background and related work

PRISM-G integrates ideas from several established areas of statistical genetics and privacy risk evaluation to detect different forms of information leakage in synthetic genomic datasets. The framework combines proximity-based disclosure testing, relatedness and haplotype structure analysis, and rare-variant–based membership inference signals.

Proximity leakage component. Detection of unusually close samples in genetic coordinate space builds on the use of principal component analysis (PCA) to represent population structure in genomic datasets [1]. PCA-derived coordinates provide interpretable axes capturing ancestry variation and have long been used for population stratification analysis. PRISM-G adapts proximity-based disclosure detection approaches proposed in the synthetic data literature [2] by testing whether holdout individuals appear in the lower tail of the synthetic–real distance distribution relative to holdout–holdout distances. To avoid falsely identifying ordinary population structure as leakage, the method incorporates an adversarial nearest-neighbor sanity check that distinguishes true proximity anomalies from normal clustering.

Kinship replay detection. Detection of replayed familial structure relies on methods developed for kinship inference and population structure analysis. Pairwise relatedness is quantified using the genetic relationship matrix (GRM), a standard representation used in quantitative genetics and mixed-model association studies [3, 4]. Excess relatedness can indicate that familial patterns present in the training data have been replicated in the synthetic dataset. PRISM-G further incorporates two complementary signals: short-range haplotype sharing detected through small genotype windows, which reflects methods used in haplotype-based relatedness and identity-by-descent (IBD) detection [5, 6, 7], and spectral diagnostics that monitor inflation of the leading eigenvalue of the GRM. Together these signals provide sensitivity to replayed family structure.

Trait-linked leakage component. Trait-linked leakage analysis builds on rare-variant burden testing and carrier probability estimation. Rare-variant burden scores aggregate the contribution of multiple uncommon variants within genomic units such as genes, a strategy used in rare variant analysis using burden testing [8]. Carrier probabilities can be approximated from allele frequencies under Hardy–Weinberg equilibrium assumptions, providing a baseline expectation for variant occurrence patterns. PRISM-G uses these ideas to construct trait-linked feature vectors and evaluate whether synthetic datasets exhibit unusual

proximity to holdout individuals in this burden space, or excess co-occurrence of rare variants that could reveal membership information.

2 Algorithm description

Given a generative model, let R_{tr} denote the real training cohort of m individuals and n single nucleotide polymorphisms (SNPs) used to produce a synthetic cohort S, and let R_{ho} denote the real holdout cohort reserved for evaluation. PRISM-G evaluates S against R_{ho} in population coordinate space, kinship space, and trait-linked feature space.

We first describe the three components used to estimate PRISM-G. Each component produces a score in $[0, 1]$, where 0 indicates no detected leakage along that axis and 1 indicates maximal exposure.

2.1 Proximity leakage index

The PLI metric evaluates whether any synthetic genome lies unusually close to a real individual in genetic coordinate space beyond what would be expected from population structure alone. For computation, real and synthetic samples are projected into a low-dimensional coordinate space using PCA of genetic variants [1]. For each synthetic sample, the distance to the nearest real individual is evaluated and compared to a reference baseline. Two complementary signals capture this behavior: **a lower-tail quantile ratio (r_p)** measuring over-closeness and an **adversarial proximity score (r_A)** reflecting nearest-neighbor concentration. These signals are combined into a normalized PLI score.

Genotypes are embedded into a d-dimensional coordinate space using principal components computed after standardizing genotypes with allele frequencies estimated from R_{tr}. Let $X_{\mathrm{ho}} \in R^{n_{\mathrm{ho}} \times d}$ and $X_S \in R^{n_S \times d}$ denote the embeddings for the holdout cohort R_{ho} and the synthetic cohort S, respectively. For each holdout individual i, define the nearest synthetic distance as

$$d_i^{\mathrm{syn}} = \min_j \|x_i^{\mathrm{ho}} - x_j^S\|_2, \tag{1}$$

and the baseline nearest real distance to other holdout samples as

$$d_i^{\mathrm{real}} = \min_{j \neq i} \|x_i^{\mathrm{ho}} - x_j^{\mathrm{ho}}\|_2. \tag{2}$$

We compare the lower tails of these distance distributions via the quantile ratio

$$\rho_q = \frac{Q_q(\{d_i^{\mathrm{syn}}\})}{Q_q(\{d_i^{\mathrm{real}}\})}, \tag{3}$$

and define the proximity signal

$$r_\rho = \text{clamp}(1 - \rho_q, 0, 1), \tag{4}$$

where $\text{clamp}(x, 0, 1) = \min\{1, \max\{0, x\}\}$.

To incorporate an adversarial check we test whether a holdout point is typically closer to another holdout sample than to any synthetic sample. Let

$$a_i = \mathbf{1}[d_i^{\text{real}} < d_i^{\text{syn}}], \qquad A = \frac{1}{n_{\text{ho}}} \sum_i a_i. \tag{5}$$

We map this to a risk score

$$r_A = \max\left\{0, \frac{0.5 - A}{0.5}\right\}. \tag{6}$$

The final PLI score is defined as

$$\text{PLI} = \max(r_\rho, r_A). \tag{7}$$

2.2 Kinship replay index

The KRI metric evaluates whether synthetic datasets reproduce patterns of relatedness present in the real cohort, such as familial relationships or population-level correlation structure. Unlike PLI, which focuses on individual proximity, KRI captures relational privacy risk that may persist even when no single synthetic genome closely matches a real individual. KRI aggregates several complementary diagnostics, each targeting a different manifestation of relatedness replay. These include reproduction of close kinship pairs, excess internal relatedness, haplotype-level collisions, and inflation of global correlation structure.

We compute GRMs for R_{ho} and S after standardizing genotypes using training-set allele frequencies following VanRaden (2008), yielding K^{ho} and K^S [4]. KRI aggregates four signals, each baseline-corrected against a safe or bootstrap reference.

First, **the replay component (r_{replay})** evaluates whether the synthetic dataset reproduces the close-kin structure observed in the real cohort. Unlike proximity-based diagnostics, this metric focuses on the relational structure of the kinship spectrum and detects whether synthetic samples recreate the distribution of close-relative relationships present in the real data.

Let K^{ho} and K^S denote the genetic relationship matrices computed for the holdout cohort R_{ho} and the synthetic cohort S, respectively. From these matrices we extract the upper-triangular kinship spectra

$$R = \{K_{ij}^{\text{ho}} : i < j\}, \qquad S = \{K_{ij}^S : i < j\}. \tag{8}$$

To focus on relatives rather than unrelated pairs, we restrict the analysis to the close-kin tail using a kinship threshold θ:

$$R_{\text{close}} = \{r \in R : r \geq \theta\}, \qquad S_{\text{close}} = \{s \in S : s \geq \theta\}. \tag{9}$$

In practice, $\theta = 0.125$ corresponds to approximately second-degree relatives.

The distributions of close-kin values are compared by constructing normalized histograms over the interval $[\theta, u]$ with B bins. Let $P_{R_{\text{close}}}$ and $P_{S_{\text{close}}}$ denote the resulting discrete distributions. The replay similarity is defined using the Jensen–Shannon divergence:

$$M = 1 - \frac{\text{JS}(P_{R_{\text{close}}}, P_{S_{\text{close}}})}{\log 2}, \tag{10}$$

where $M \in [0, 1]$ measures the similarity between the real and synthetic close-kin spectra.

To estimate the similarity expected under a null model, bootstrap samples are generated by resampling synthetic kinship values with replacement. Let $S^{(b)}$ denote the b-th bootstrap replicate and $M^{(b)}$ the corresponding replay similarity. The null expectation is

$$M_0 = E_b[M^{(b)}]. \tag{11}$$

The replay risk signal is then defined as

$$r_{\text{replay}} = \text{clamp}\left(\frac{M - M_0}{1 - M_0}, \, 0, \, 1\right), \tag{12}$$

where M is the observed replay rate and M_0 is the permutation baseline replay rate.

Second, **the internal kinship excess (r_{IKE})** component evaluates whether the synthetic dataset exhibits an overall surplus of relatedness relative to the real cohort. While the replay metric focuses on reproducing close-kin pairs, r_{IKE} tests whether synthetic samples are mutually related more often than expected under the baseline kinship distribution.

Given the synthetic kinship matrix K^S, we define the fraction of synthetic pairs whose kinship exceeds a threshold θ as

$$f(\theta) = \frac{\#\{(i, j) : K^S_{ij} \geq \theta, \, i < j\}}{\binom{n_S}{2}}. \tag{13}$$

To estimate the expected baseline fraction of related pairs, we use bootstrap resampling from the holdout kinship spectrum. Let K^{ho} denote the holdout kinship matrix and sample $\binom{n_S}{2}$ values with replacement from its upper-triangular entries. For each bootstrap replicate b, we compute

$$f_0^{(b)}(\theta) = \frac{1}{m} \sum_{k=1}^{m} \mathbf{1}\left[K_0^{(b)}(k) \geq \theta\right], \tag{14}$$

where m is the number of sampled kinship values and $\mathbf{1}[\cdot]$ denotes the indicator function. Repeating this procedure B times yields the expected baseline fraction

$$f_0(\theta) = \frac{1}{B}\sum_{b=1}^{B} f_0^{(b)}(\theta). \tag{15}$$

The internal kinship excess signal is then defined as the normalized surplus above this baseline:

$$r_{\mathrm{IKE}} = \mathrm{clamp}\left(\frac{f(\theta) - f_0(\theta)}{1 - f_0(\theta)},\, 0,\, 1\right), \tag{16}$$

where $f(\theta)$ is the observed fraction of pairs in the synthetic dataset with kinship coefficient at least θ, and $f_0(\theta)$ is the corresponding baseline fraction estimated from the reference distribution.

Third, **the micro haplotype collision (**r_{HAP}**)** detects excess reuse of short genotype patterns across synthetic genomes. Short haplotype segments can reveal unintended duplication of genetic structure [6], even when overall proximity and kinship signals appear benign. To quantify this effect, we compute windowed haplotype collisions across the synthetic genotype matrix.

For a window of k consecutive SNPs, we extract the genotype submatrix and encode each individual's genotype vector as a discrete haplotype key. Let n_u denote the number of samples sharing haplotype pattern u within that window. The number of colliding haplotype pairs in window w is defined as

$$c_w = \sum_u \frac{n_u(n_u - 1)}{2}. \tag{17}$$

The per-window collision rate is then

$$\rho_w = \frac{c_w}{\binom{n}{2}}, \tag{18}$$

where n is the number of synthetic samples. Let $c_{\max} = \max_w \rho_w$ denote the maximum collision rate across windows. The overall collision rate is obtained by averaging across all W windows:

$$c = \frac{1}{W}\sum_w \rho_w. \tag{19}$$

To estimate the baseline expectation, the same statistic is computed on the holdout genotype matrix over bootstrap replicates, yielding

$$c_0 = \frac{1}{B}\sum_{b=1}^{B} c_0^{(b)}. \tag{20}$$

The micro-haplotype collision signal is then defined as the normalized excess above this baseline:

$$r_{\mathrm{HAP}} = \frac{c - c_0}{c_{\max} - c_0}, \tag{21}$$

where c is the observed collision rate, c_0 is the baseline collision rate, and $c_{\max}$ is the maximum achievable collision rate under the chosen window configuration.

Fourth, **the spectral inflation (r_{SPEC})** component detects abnormal concentration of relatedness in the synthetic kinship matrix. Such concentration may arise when latent clusters or duplicated structure are reproduced in the synthetic cohort. Given the synthetic kinship matrix K^S, we approximate its largest eigenvalue λ_1 using power iteration:

$$v_{t+1} = \frac{K^S v_t}{\|K^S v_t\|}, \qquad \lambda_1 = \lim_{t\to\infty} \frac{(K^S v_t)\cdot v_t}{v_t \cdot v_t}. \tag{22}$$

We then compute the normalized leading-eigenvalue share

$$s = \frac{\lambda_1}{\text{trace}(K^S)}, \tag{23}$$

where $\text{trace}(K^S) = \sum_i K^S_{ii}$ represents the total genetic variance across individuals. Large values of s indicate that a single eigenmode captures a disproportionate share of the variance, reflecting potential over-clustering or replayed relatedness structure.

To derive the baseline expectation, the same statistic is computed on the holdout kinship matrix K^{ho}:

$$s_0 = \frac{\lambda_1(K^{\text{ho}})}{\text{trace}(K^{\text{ho}})}. \tag{24}$$

The spectral inflation signal is then defined as

$$r_{\text{SPEC}} = 1 - \frac{s_0}{s}, \tag{25}$$

where s is the observed spectral statistic and s_0 is the corresponding baseline value.

Finally, the KRI score aggregates the four relatedness signals as

$$\text{KRI} = \max\left(r_{\text{replay}}, r_{\text{IKE}}, r_{\text{HAP}}, r_{\text{SPEC}}\right). \tag{26}$$

2.3 Trait-linked linkage index

The TLI metric captures privacy risk arising from genetic features associated with phenotypes or distinctive individual characteristics, with a particular focus on rare genetic variants. Such features may enable attribute inference or membership attacks even in the absence of close genetic similarity. We quantify TLI using two complementary signals: **the performance of a simple membership inference attack (r_{mia})** and **the presence of rare-variant collisions (r_{uniq}) between synthetic and real individuals**. These terms capture different mechanisms by which trait-linked information may be exposed and are combined into a normalized TLI score as described below.

Let c_i denote the trait-linked feature vector for individual i, representing estimated rare-variant burden scores that aggregate the contribution of rare variants within predefined genomic units (e.g., chromosome sets) [8]. To assess whether the presence of training individuals leaves a detectable trace in the synthetic cohort S, we compute distances between each real individual and the synthetic set in the trait-feature space. For each individual i, we define the nearest synthetic distance

$$d_S(i) = \min_{s \in S} \|c_i - c_s\|. \tag{27}$$

Using the synthetic cohort as reference, we assign membership scores to real individuals as

$$s(i) = -d_S(i), \tag{28}$$

so that smaller distances correspond to stronger membership evidence. Training individuals are labeled as 1 and holdout individuals as 0. We then evaluate the area under the receiver operating characteristic curve (ROC AUC), denoted by A, and define the membership inference advantage as

$$r_{\text{mia}} = \max\left\{0, \frac{0.5 - A}{0.5}\right\} \tag{29}$$

so that random performance maps to 0 and perfect separation maps to 1.

The uniqueness and rarity match component evaluates whether rare variant signals occur more frequently in the synthetic dataset than expected under a null model driven by training allele frequencies [9]. Let the rare-variant set be defined as

$$R = \{j : \text{MAF}_j^{\text{train}} < \tau\}.$$

For SNP j with training allele frequency p_j, under a Hardy–Weinberg equilibrium approximation the diploid carrier probability is

$$q_j \approx 2p_j.$$

Given a synthetic cohort of size n_S, let $X_j \sim \text{Binomial}(n_S, q_j)$ denote the number of synthetic individuals carrying at least one copy of the variant. The probability that at least two individuals carry the variant under the null model is

$$\Pr[X_j \geq 2] = 1 - (1 - q_j)^{n_S} - n_S q_j (1 - q_j)^{n_S - 1}. \tag{30}$$

Averaging this probability across all rare variants yields the expected baseline fraction

$$U_0 = \frac{1}{|R|} \sum_{j \in R} \Pr[X_j \geq 2].$$

From the synthetic dataset we compute the observed fraction of rare variants with at least two carriers

$$U = \frac{1}{|R|} \sum_{j \in R} \mathbf{1}[X_j \geq 2]. \tag{31}$$

The rare-variant uniqueness signal is then defined as

$$r_{\text{uniq}} = \text{clamp}\left(\frac{U - U_0}{1 - U_0},\, 0,\, 1\right). \tag{32}$$

Finally, the TLI metric aggregates the two trait-linked signals as

$$\text{TLI} = \max\left(r_{\text{mia}}, r_{\text{uniq}}\right). \tag{33}$$

2.4 Aggregation of component risks

Each component produces a unit-interval risk score with higher values indicating greater privacy risk: PLI for coordinate proximity, KRI for kinship replay, and TLI for trait-linked exposure. Because raw component scores are not directly comparable across datasets or generators, we aggregate them into a single cohort-level risk score. To do so, we use a risk-averse, OR-like aggregation that preserves monotonicity and ensures that a single high-risk component cannot be masked by lower values in the others.

Specifically, let $r_{\text{PLI}}, r_{\text{KRI}}, r_{\text{TLI}} \in [0, 1]$ denote the component scores and $w_{\text{PLI}}, w_{\text{KRI}}, w_{\text{TLI}} \geq 0$ weights that sum to one. The aggregated raw risk is defined as

$$R_{\text{raw}} = 1 - \prod_{k \in \{\text{PLI},\text{KRI},\text{TLI}\}} \left(1 - w_k r_k\right). \tag{34}$$

This construction has three practical advantages. First, it is monotone in every component: increasing any r_k increases R_{raw}. Second, it is risk-averse: if any component approaches one, R_{raw} also approaches one regardless of the others. Third, it is scale-free with respect to cohort size, since all component scores are normalized to the unit interval.

When a sub-diagnostic within KRI is not applicable (for example, the replay term when the holdout set contains no close relatives), that term is omitted from KRI's internal maximum without renormalizing the remaining components. This preserves comparability across datasets.

2.5 Calibration and estimation of weights

Because raw risk scores are not directly comparable across datasets or generators, PRISM-G uses simple reference generators to anchor the risk scale. This calibration step ensures that scores are interpretable and comparable without assuming a specific adversary model. We calibrate R_{raw} using two reference generators that anchor the lower and upper ends of the scale:

- A **safe baseline** (A_{Safe}) that preserves allele frequencies but intentionally removes dependence structure (e.g., an allele-frequency–matched marginal sampler).
- One or more **leaky baselines** (A_{Leaky}) that intentionally overfit structure (e.g., copycat or kinship-preserving generators).

To prevent the estimator from concentrating weight on a single component and artificially inflating overall risk, we calibrate the component weights using a ridge-regularized fit to the anchor baselines. Specifically, we choose weights w that match target risks at the anchors while remaining close to equal weights w_0:

$$\min_{w \in \Delta} \left(R(A_{\text{Safe}}; w) - \varepsilon\right)^2 + \left(R(A_{\text{Leaky}}; w) - \lambda\right)^2 + \gamma \|w - w_0\|_2^2, \tag{35}$$

subject to $w_m \geq 0$ and $\sum_m w_m = 1$.

This calibration introduces three hyperparameters controlling the scale of R_{raw}. The safe target ε anchors $R(A_{\text{Safe}}; w)$ to a small band (0.01–0.1), avoiding degenerate solutions that force component weights toward zero. The leaky target λ (0.75–0.90) anchors $R(A_{\text{Leaky}}; w)$ near the upper end of the scale while leaving headroom for more extreme cases. Finally, the penalty strength γ (10^{-4}–10^{-2}) shrinks the weights toward the prior w_0, balancing anchor fit with stability and discouraging single-component dominance.

2.6 Estimation of PRISM-G score and statistical ranking

For each baseline generator we compute the distribution of R_{raw}. Let α denote the calibrated lower anchor of R_{raw} under the safe baseline, and β the upper anchor under the leaky baseline. The observed raw risk for a model of interest is then mapped to a standardized 0–100 scale as

$$\text{PRISM-G} = 100 \times \frac{R_{\text{raw}} - \alpha}{\beta - \alpha}. \tag{36}$$

This linear calibration is monotone and model-agnostic, producing scores that remain comparable across datasets provided the same anchor generators are used.

To assess the robustness of the resulting rankings, we evaluate stability using Kendall's rank correlation coefficient. Using bootstrap resampling, let $\pi^{(b)}$ denote the ranking of generators at bootstrap replicate b, and let $\pi^{(0)}$ denote the baseline ranking. The stability statistic is defined as

$$\bar{\tau} = \frac{1}{B} \sum_{b=1}^{B} \tau\left(\pi^{(b)}, \pi^{(0)}\right), \tag{37}$$

where $\bar{\tau}$ is the mean Kendall rank coefficient and B is the number of bootstrap replicates. A permutation p-value is obtained by comparing $\bar{\tau}$ with the distribution of $\tau(\pi^{(\text{perm})}, \pi^{(0)})$ under random permutations of the rankings.

2.7 Grid-search evaluation

Finally, for hyperparameter tuning, we perform a grid search over the calibration parameters to maximize $\bar{\tau}$ for PRISM-G score estimation. Given parameter grids $\varepsilon \in \mathcal{E}$, $\lambda \in \Lambda$, and $\gamma \in \Gamma$, we evaluate each triple $(\varepsilon, \lambda, \gamma)$ and select the configuration that yields the highest $\bar{\tau}$. The procedure is summarized in Algorithm 1.

Algorithm 1 Grid search for PRISM-G calibration

Require: Parameter grids $\varepsilon \in \mathcal{E}$, $\lambda \in \Lambda$, $\gamma \in \Gamma$
Ensure: Optimal calibration parameters $(\varepsilon^*, \lambda^*, \gamma^*)$

```
1: for each (ε, λ, γ) ∈ 𝓔 × Λ × Γ do
2:     Fit weights w* using the objective function in Eq. (17)
3:     Compute PRISM-G scores for all candidate datasets
4:     Bootstrap anchors to obtain ranking samples and compute τ̄
5: end for
6: Select (ε*, λ*, γ*) = arg max τ̄
7: return (ε*, λ*, γ*)
```

3 Evaluation procedure

3.1 Generative models

We evaluated PRISM-G on synthetic cohorts generated using three model families: (1) generative adversarial networks (GANs), (2) restricted Boltzmann machines (RBMs) as introduced by Yelmen et al. (2021) [10], and (3) Genomator, a logic-based generator that formulates genome synthesis as a satisfiability (SAT) problem [11].

GANs consist of a generator that maps random noise to genotype vectors and a discriminator that distinguishes real from synthetic samples. Through adversarial training, the generator learns to reproduce key genomic properties such as allele frequencies, population structure, and pairwise correlations; conditional or convolutional layers can further control ancestry and improve scalability. RBMs learn the joint distribution of genotype vectors through an energy-based model. After training (e.g., via contrastive divergence), synthetic genomes are generated through alternating Gibbs updates, allowing the model to capture linkage disequilibrium and higher-order dependencies in the data.

In contrast, Genomator encodes admissible genotype configurations as logical constraints and uses SAT solving to generate synthetic genomes that satisfy specified biological and privacy rules. A tunable parameter controls genetic distances both to the training data and within the synthetic cohort.

As a reference dataset, we used the Phase 3 release of the 1000 Genomes Project (1KGP), comprising 2,504 individuals [12]. This dataset served as the source for training and holdout partitions and provided a common marker set for all experiments.

3.2 Synthetic datasets

For the GAN and RBM baselines, we used the synthetic cohorts released by Yelmen et al. (2023), generated from 1KGP. For Genomator, we generated new synthetic cohorts from the same training splits under three Hamming distance settings $H \in \{1, 10, 50\}$, which regulate proximity between training and synthetic samples as well as among synthetic individuals.

All analyses were conducted on two SNP panels: a chromosome 15 dataset containing 10,000 SNPs and a chromosome 1 dataset containing 65,535 SNPs. For each dataset and generator, the size of the synthetic cohort matched the corresponding holdout cohort.

3.3 Parameters Settings

For PRISM-G evaluation, each dataset was split into training (80%) and holdout (20%) sets. The training set was used to estimate baseline statistics for the component metrics and to compute embeddings, whereas the holdout set was used for final estimation of component scores and membership-inference evaluation. Component metrics were estimated using 1,000 bootstrap replicates.

For PLI, genetic coordinates were obtained by performing PCA on the training genotypes and retaining the first 10 components. The proximity diagnostic used a fixed lower-tail quantile of $q = 0.01$ in the distance quantile ratio to focus on the closest real–synthetic pairs.

For KRI, the replay threshold was set to 0.125 (approximately first-cousin relatedness), with tolerance $\tau = 0.01$ when matching close-kin edges between real and synthetic cohorts. The internal kinship excess (r_{IKE}) was evaluated at thresholds $\theta \in \{0.10, 0.125, 0.25\}$, and KRI retained the maximum excess across these levels.

For TLI, rare variants were defined by minor allele frequency (MAF) < 0.001, with allele frequencies estimated from the training set. Membership inference risk was computed as described in the PRISM-G methodology.

For the initial estimation of raw scores, component weights were calibrated using $\varepsilon = 0.02$, $\lambda = 0.75$, and $\gamma = 10^{-3}$, and ranking stability was evaluated over 1,000 bootstrap replicates. For hyperparameter tuning, we performed a grid search over $\varepsilon \in \{0.02, 0.04, 0.06, 0.08, 0.10\}$, $\lambda \in \{0.75, 0.80, 0.85, 0.90\}$, and $\gamma \in \{10^{-4}, 10^{-3}, 10^{-2}\}$, evaluating each configuration with 1,000 bootstrap replicates.

3.4 Calibration anchors

We used two reference generators to calibrate PRISM-G. The safe baseline is constructed using a binomial sampler. For each SNP j with training-set allele frequency p_j^{train}, and for each synthetic individual i, we draw an independent diploid genotype

$$X_{ij} \sim \mathrm{Binomial}(2, p_j^{\mathrm{train}}), \qquad X_{ij} \in \{0, 1, 2\}. \tag{38}$$

Thus,

$$\Pr[X_{ij} = g] = \binom{2}{g} (p_j^{\text{train}})^g (1 - p_j^{\text{train}})^{2-g}, \qquad g \in \{0, 1, 2\}. \tag{39}$$

Genotypes are drawn independently across individuals and SNPs using the same marker set and sample size as the holdout cohort. This construction preserves site-level allele frequencies ($E[X_{ij}/2] = p_j^{\text{train}}$) while removing linkage disequilibrium, kinship structure, and trait-linked correlations.

The leaky baseline is a copycat, kin-doped reference that intentionally over-preserves structure. Each synthetic genome X_i is templated from a training individual $t(i)$ with a small per-site perturbation (e.g., Hamming distance $H(X_i, X_{t(i)}) \leq \delta$), and a subset of templates is duplicated or paired to induce close-kin edges with target kinship $\theta \in [0.125, 0.25]$. This procedure produces synthetic cohorts unusually close to training genomes and enriched for relatedness, serving as a high-risk anchor on the PRISM-G scale.

3.5 Utility evaluation and privacy–utility trade-off

To evaluate the privacy–utility trade-off of synthetic genomic datasets, we assessed their performance in a genetic ancestry inference task. Population structure was first estimated from the real training cohort using principal component analysis (PCA). The first ten principal components were retained to define the genetic coordinate space used for downstream analyses. Synthetic genomes were then projected onto the same PCA embedding to ensure comparability between real and synthetic samples.

Utility was evaluated using the superpopulation labels provided by the 1KGP dataset, including African (AFR), European (EUR), East Asian (EAS), South Asian (SAS), and Admixed American (AMR) populations. A random forest classifier was trained on the real training samples using the ten principal components and the corresponding superpopulation labels, and then applied to the synthetic datasets to predict ancestry assignments.

We quantified population-structure preservation using two complementary metrics. First, Procrustes similarity was computed between the centroids of real and synthetic ancestry clusters in the ten-dimensional PCA space, providing a measure of geometric alignment between population structures. Second, we measured the JS similarity between the distributions of predicted ancestry labels in the synthetic data and the corresponding distributions in the real holdout data. JS similarity was calculated using the proportions of individuals assigned to each superpopulation group.

Part II
Supplementary Results

Table 1: Proximity leakage index (PLI) submetrics for the 10,000 SNPs panel dataset (chromosome 15).

Model	ρ_q	r_ρ	A_{adv}	r_A
GAN	1.053	0.000	0.349	0.301
RBM	1.099	0.000	0.327	0.345
Genomator (H = 1)	0.191	0.809	0.086	0.828
Genomator (H = 10)	0.412	0.588	0.096	0.808
Genomator (H = 50)	0.738	0.262	0.134	0.733

Table 2: Kinship replay index (KRI) submetrics for the 10,000 SNPs panel dataset (chromosome 15).

Model	r_{replay}	r_{IKE}	r_{HAP}	r_{SPEC}
GAN	0.000	0.266	0.008	0.435
RBM	0.001	0.000	0.032	0.000
Genomator (H = 1)	0.003	0.000	0.013	0.750
Genomator (H = 10)	0.026	0.020	0.018	0.789
Genomator (H = 50)	0.022	0.057	0.043	0.813

Table 3: Trait linkage index (TLI) submetrics for the 10,000 SNPs panel dataset (chromosome 15).

Model	MIA_{AUC}	r_{mia}	U	U_0	r_{uniq}
GAN	0.483	0.000	0.664	0.539	0.271
RBM	0.500	0.001	0.905	0.539	0.794
Genomator (H = 1)	0.520	0.039	0.008	0.538	0.000
Genomator (H = 10)	0.512	0.024	0.005	0.538	0.000
Genomator (H = 50)	0.518	0.035	0.003	0.538	0.000

Table 4: Proximity leakage index (PLI) submetrics for the 65,535 SNPs panel dataset (chromosome 1).

Model	ρ_q	r_ρ	A_{adv}	r_A
GAN	1.230	0.000	0.723	0.000
RBM	1.259	0.000	0.806	0.000
Genomator (H = 1)	0.569	0.431	0.323	0.353
Genomator (H = 10)	0.817	0.183	0.421	0.158
Genomator (H = 50)	0.949	0.052	0.523	0.000

Table 5: KRI submetrics for the 65,535 SNP panel dataset (chromosome 1).

Model	r_{replay}	r_{IKE}	r_{HAP}	r_{SPEC}
GAN	0.000	0.000	0.000	0.541
RBM	0.002	0.000	0.000	0.000
Genomator (H = 1)	0.000	0.000	0.034	0.441
Genomator (H = 10)	0.000	0.023	0.055	0.488
Genomator (H = 50)	0.000	0.093	0.082	0.560

Table 6: TLI submetrics for the 65,535 SNP panel dataset (chromosome 1).

Model	MIA_{AUC}	r_{mia}	U	U_0	r_{uniq}
GAN	0.502	0.003	0.636	0.719	0.000
RBM	0.514	0.028	0.875	0.719	0.555
Genomator (H = 1)	0.535	0.070	0.026	0.718	0.000
Genomator (H = 10)	0.526	0.052	0.019	0.718	0.000
Genomator (H = 50)	0.540	0.079	0.021	0.718	0.000

Table 7: PRISM-G scores and metrics for calibration anchors (10,000 SNP panel dataset).

Model	PLI	KRI	TLI	α/β	PRISM-G
Safe binomial sampler	0.005	0.108	0.012	0.008	0.000
Leaky copycat	1.000	0.206	1.000	0.748	100.000

Table 8: PRISM-G scores and metrics for calibration anchors (65,535 SNP panel dataset).

Model	PLI	KRI	TLI	α/β	PRISM-G
Safe binomial sampler	0.000	0.000	0.019	0.013	0.000
Leaky copycat	1.000	0.003	1.000	0.749	100.000

Table 9: PRISM-G components for the 10,000 SNP panel dataset (chromosome 15).

Model	PLI	KRI	TLI	R_{raw}
GAN	0.301	0.435	0.271	0.272
RBM	0.345	0.018	0.794	0.568
Genomator (H = 1)	0.828	0.750	0.039	0.269
Genomator (H = 10)	0.808	0.789	0.024	0.261
Genomator (H = 50)	0.733	0.813	0.035	0.254

Table 10: PRISM-G scores for the 10,000 SNP panel dataset (chromosome 15; default hyperparameters; 1,000 bootstraps).

Model	PRISM-G	SD	LCI (95%)	UCI (95%)
RBM	70.368	8.920	47.738	77.311
Genomator (H = 1)	45.531	15.041	31.758	80.729
Genomator (H = 10)	44.104	14.842	30.976	79.199
Genomator (H = 50)	41.576	13.102	31.074	73.304
GAN	35.312	1.070	33.589	37.970

Table 11: PRISM-G scores for the 65,535 SNP panel dataset (chromosome 1; default hyperparameters; 1,000 bootstraps).

Model	PRISM-G	SD	LCI (95%)	UCI (95%)
RBM	42.763	7.404	22.677	50.267
Genomator (H = 1)	26.534	4.638	22.144	39.300
Genomator (H = 10)	13.756	1.669	11.881	18.542
Genomator (H = 50)	10.111	2.615	6.065	14.816
GAN	2.102	2.223	0.000	6.682

Table 12: Kendall rank stability results for PRISM-G across bootstrap replicates (default hyperparameters).

Dataset	$\bar{\tau}$	SD	LCI (95%)	UCI (95%)	P-value
10,000 SNPs (Chromosome 15)	0.699	0.287	0.200	1.000	0.074
65,535 SNPs (Chromosome 1)	0.962	0.081	0.800	1.000	0.018

Table 13: PRISM-G scores for the 10,000 SNP panel dataset (chromosome 15; grid search results).

Model	PRISM-G	SD	LCI (95%)	UCI (95%)
RBM	69.761	5.832	56.027	74.412
Genomator (H = 1)	48.910	10.372	39.747	71.715
Genomator (H = 10)	47.313	10.259	38.332	69.986
Genomator (H = 50)	44.165	9.080	36.369	64.486
GAN	35.392	0.716	34.091	36.944

Table 14: PRISM-G scores and metrics for the 65,535 SNP panel dataset (chromosome 1; grid search results).

Model	PRISM-G	SD	LCI (95%)	UCI (95%)
RBM	42.034	3.837	30.477	45.554
Genomator (H = 1)	27.535	2.840	24.594	36.179
Genomator (H = 10)	13.847	1.153	12.008	17.148
Genomator (H = 50)	9.628	1.089	7.159	11.239
GAN	1.204	0.880	0.000	2.740

Table 15: Optimal PRISM-G calibration parameters and Kendall rank stability statistics obtained from the grid search procedure.

Dataset	ϵ	λ	γ	$\bar{\tau}$	SD	LCI (95%)	UCI (95%)	P-value
10,000 SNPs (Chromosome 15)	0.04	0.75	0.01	0.893	0.219	0.400	1.000	0.018
65,535 SNPs (Chromosome 1)	0.04	0.75	0.01	0.985	0.052	0.800	1.000	0.018

Table 16: Permutation test for the 10,000 SNP panel dataset (chromosome 15; grid search results).

Model comparison	Mean diff.	LCI (95%)	UCI (95%)	P-value
GAN - RBM	-34.370	-39.491	-19.395	0.00
GAN - Genomator (H = 1)	-13.519	-35.084	-4.658	0.00
GAN - Genomator (H = 10)	-11.922	-33.348	-3.309	0.00
Genomator (H = 1) - Genomator (H = 10)	1.597	1.357	1.855	0.00
Genomator (H = 10) - Genomator (H = 50)	3.149	1.863	5.554	0.00
Genomator (H = 1) - Genomator (H = 50)	4.746	2.329	7.319	0.00
GAN - Genomator (H = 50)	-8.773	-27.825	-1.458	0.00
RBM - Genomator (H = 50)	25.597	-8.413	37.644	2.82E-01
RBM - Genomator (H = 10)	22.448	-13.933	35.616	3.88E-01
RBM - Genomator (H = 1)	20.851	-15.668	34.185	3.96E-01

Table 17: Permutation test for the 65,535 SNP panel dataset (chromosome 1; grid search results).

Model comparison	Mean diff.	LCI (95%)	UCI (95%)	P-value
GAN - RBM	-40.830	-43.958	-29.980	0.00
GAN - Genomator (H = 1)	-26.331	-34.869	-22.702	0.00
GAN - Genomator (H = 10)	-12.643	-15.969	-11.287	0.00
GAN - Genomator (H = 50)	-8.424	-8.717	-7.159	0.00
RBM - Genomator (H = 10)	28.186	13.949	32.206	0.00
RBM - Genomator (H = 50)	32.406	21.874	35.397	0.00
Genomator (H = 1) - Genomator (H = 10)	13.687	11.416	19.031	0.00
Genomator (H = 1) - Genomator (H = 50)	17.907	14.069	26.905	0.00
Genomator (H = 10) - Genomator (H = 50)	4.219	2.654	7.921	0.00
RBM - Genomator (H = 1)	14.499	-5.065	20.340	1.46E-01

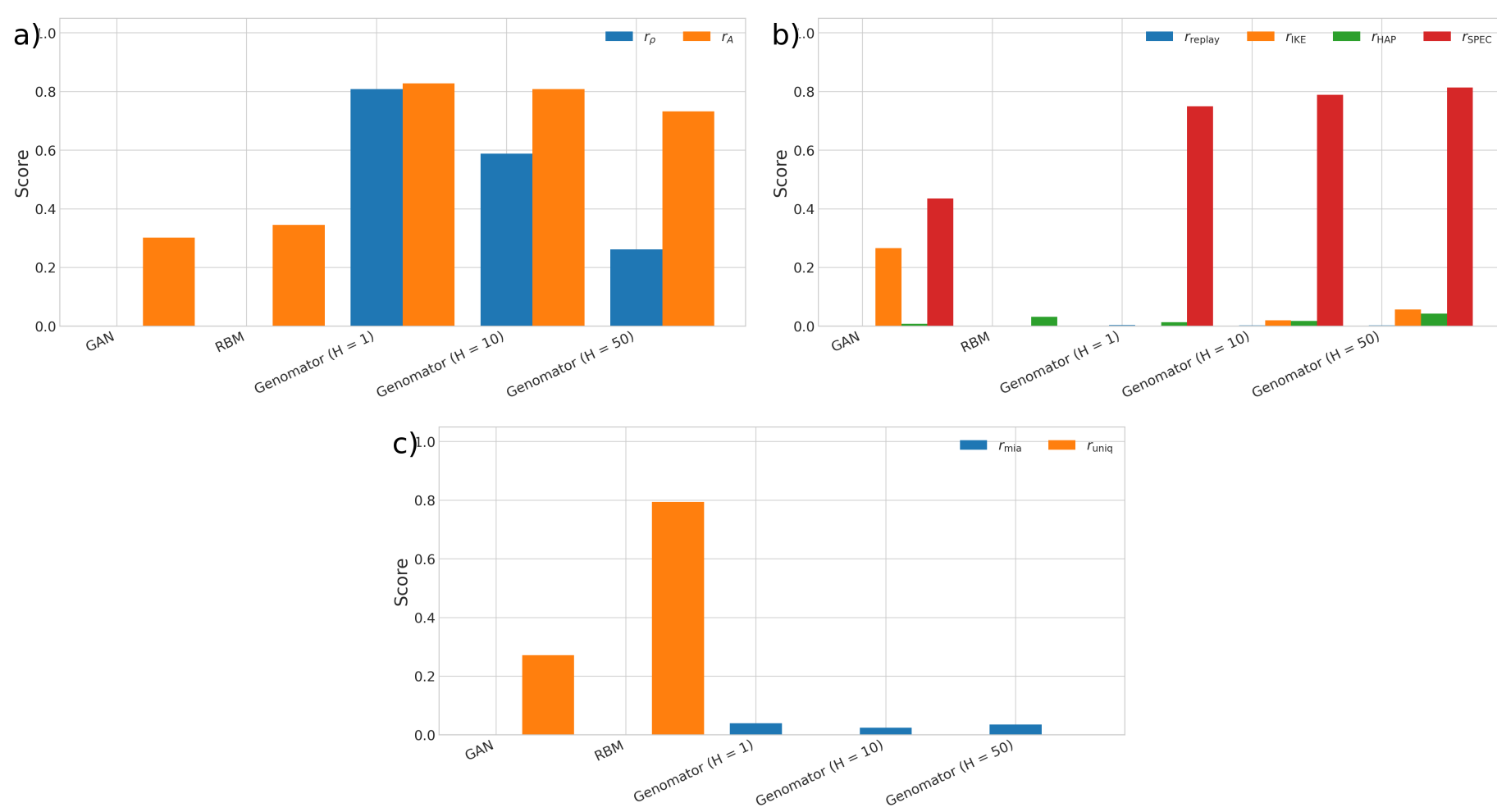

Figure 1: PRISM-G submetrics for the 10,000 SNPs panel dataset. Each barplot shows a) Proximity leakage components: proximity quantile and adversarial distance signals. b) Kinship replay components: replay, internal kinship excess, micro haplotype collision, and spectral anomaly. c) Trait leakage components: membership-inference performance, uniqueness and rarity match.

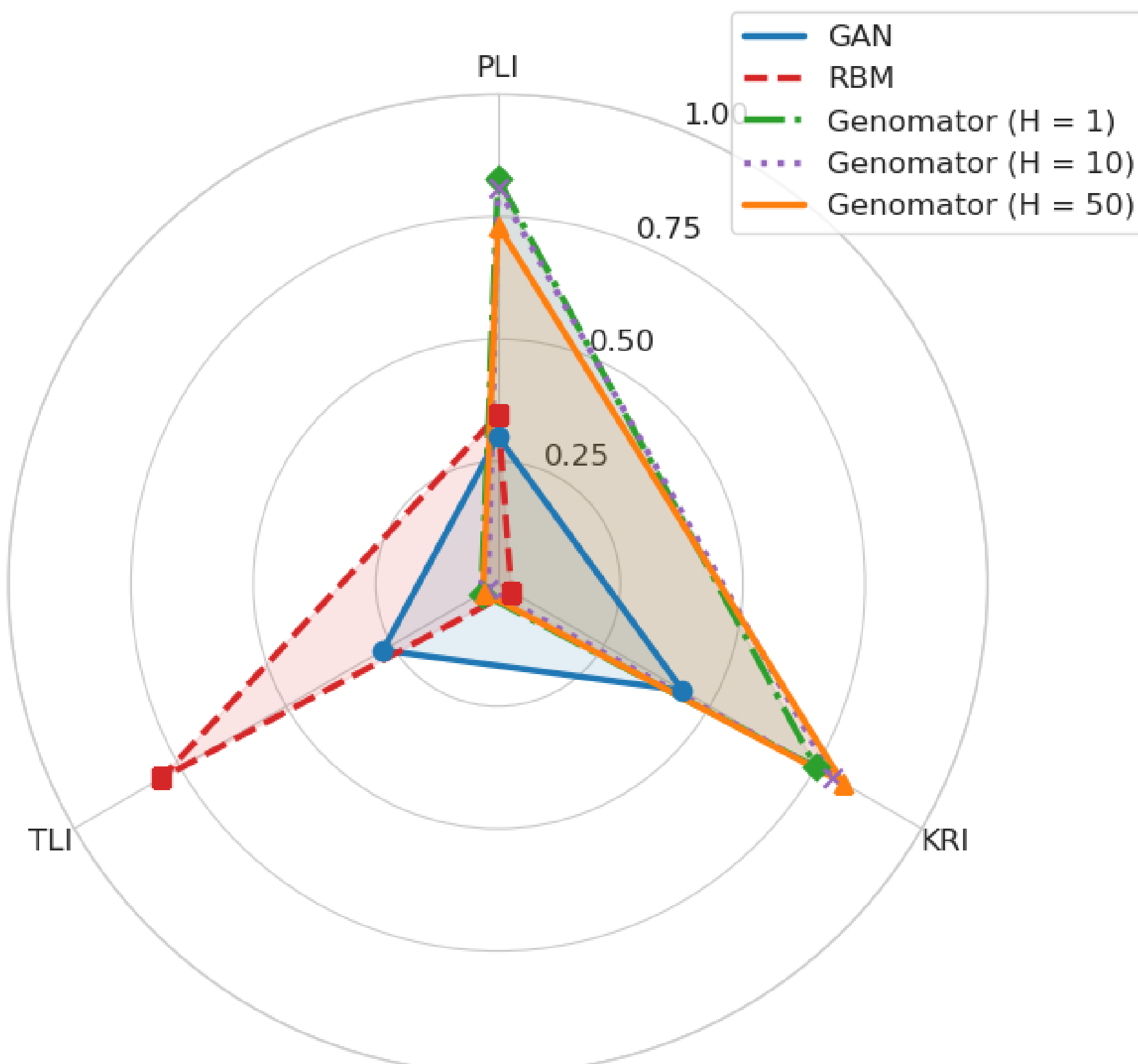

Figure 2: PRISM-G components for the 10,000 SNPs panel dataset.

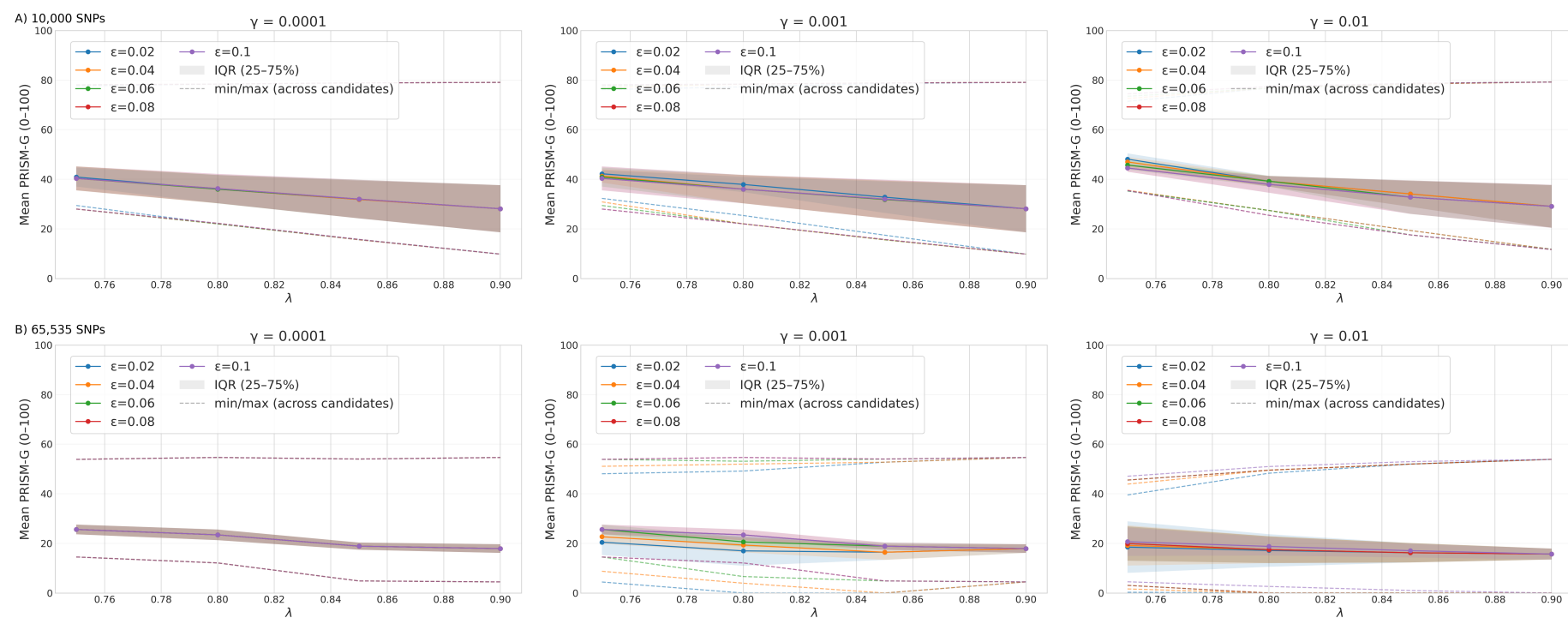

Figure 3: Sensitivity analysis to assess the effect of calibration hyperparameters on PRISM-G scores. Mean PRISM-G scores are shown as a function of the calibration parameter λ for different values of ϵ. Shaded regions indicate the interquartile range of PRISM-G scores across bootstrap replicates, while dashed horizontal lines denote the minimum and maximum scores observed across replicates and hyperparameter settings. Panels show results for (a) the 10,000 SNP panel dataset and (b) the 65,535 SNP panel dataset. From left to right, plots correspond to $\gamma \in \{10^{-4}, 10^{-3}, 10^{-2}\}$.

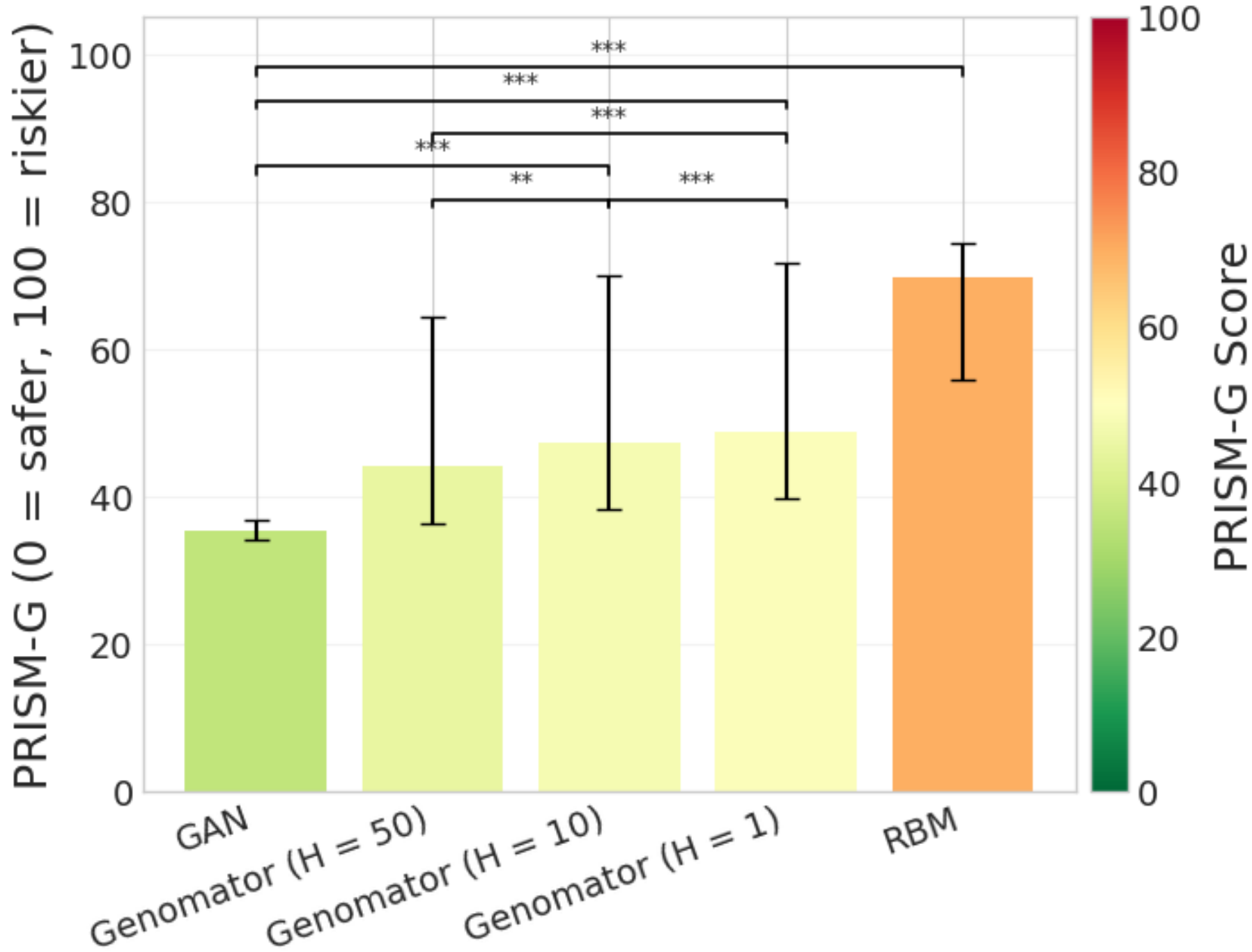

Figure 4: Mean PRISM-G scores for synthetic data generators and pairwise comparisons using the optimal model hyperparameters obtained using grid search. 10,000 SNPs panel datasets

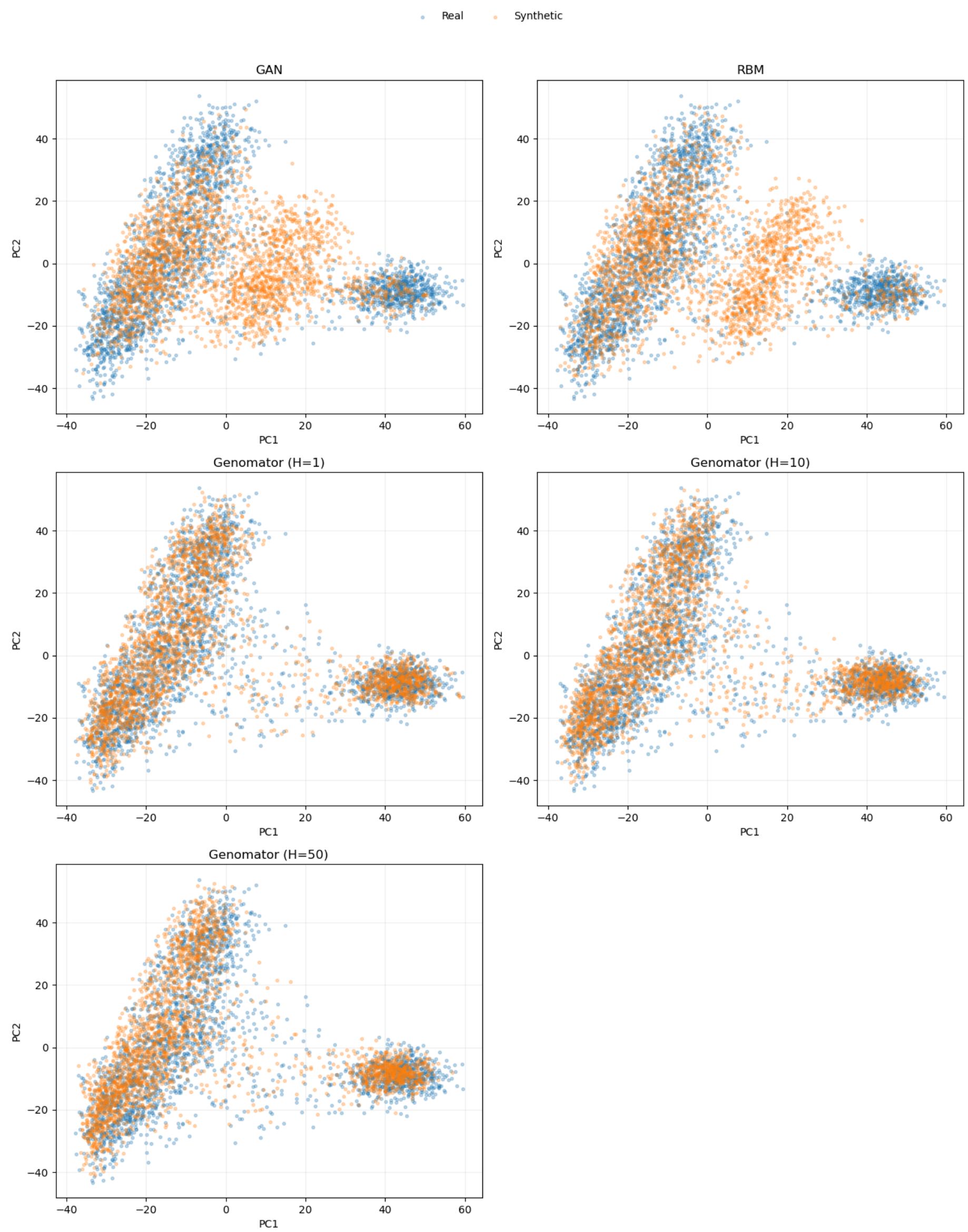

Figure 5: PCA of real and synthetic genomes (Chromosome 15; 10,000 SNPs). PCA was fitted on the real dataset and synthetic samples were projected onto the same PCA space. Blue points represent real samples and orange points represent synthetic samples generated by different models (GAN, RBM, and Genomator with $H = 1, 10, 50$). The overlap indicates how well each model reproduces the population structure.

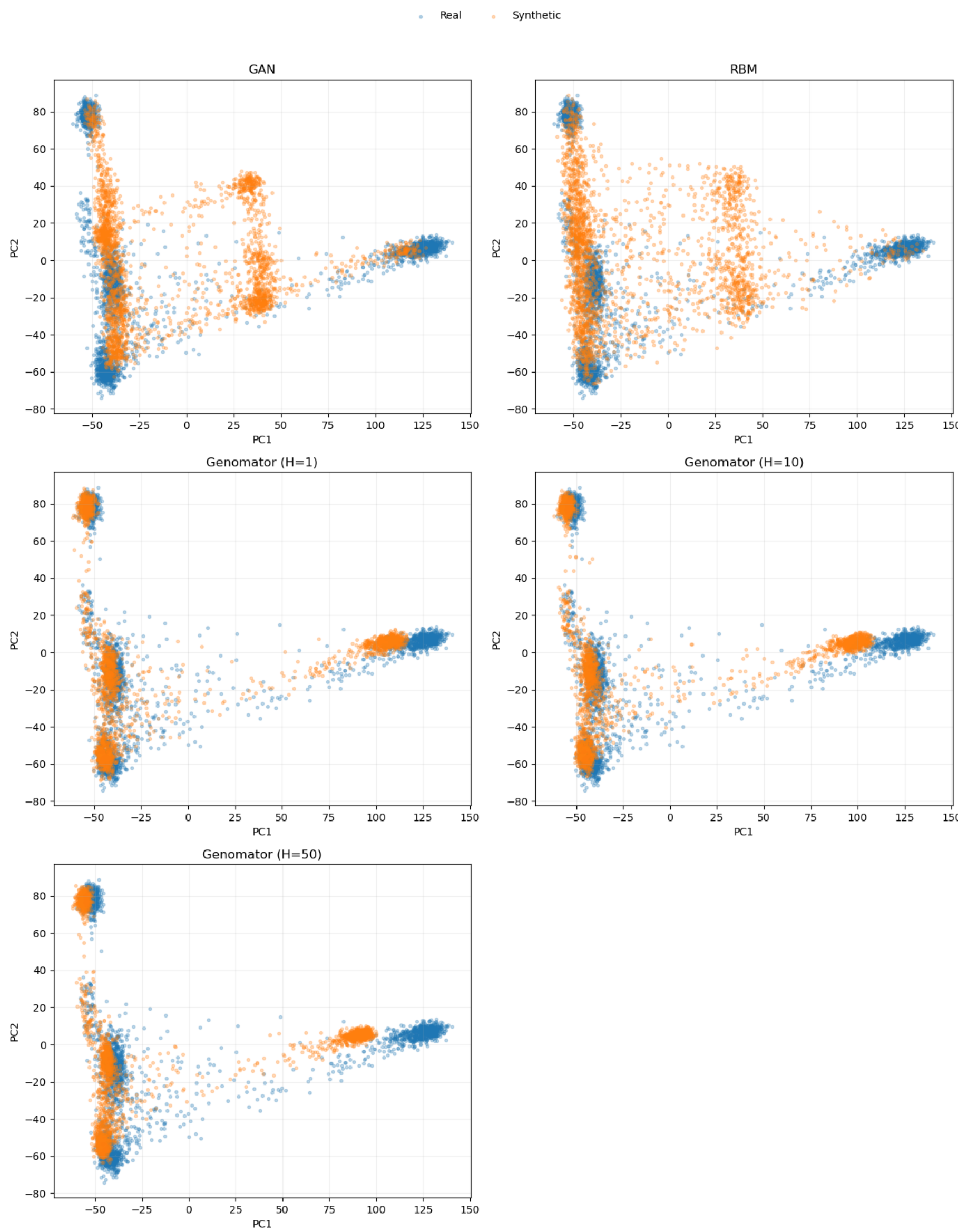

Figure 6: PCA of real and synthetic genomes (Chromosome 1; 65,535 SNPs). PCA was fitted on the real dataset and synthetic samples were projected onto the same PCA space. Blue points represent real samples and orange points represent synthetic samples generated by different models (GAN, RBM, and Genomator with $H = 1, 10, 50$). The overlap indicates how well each model reproduces the population structure.

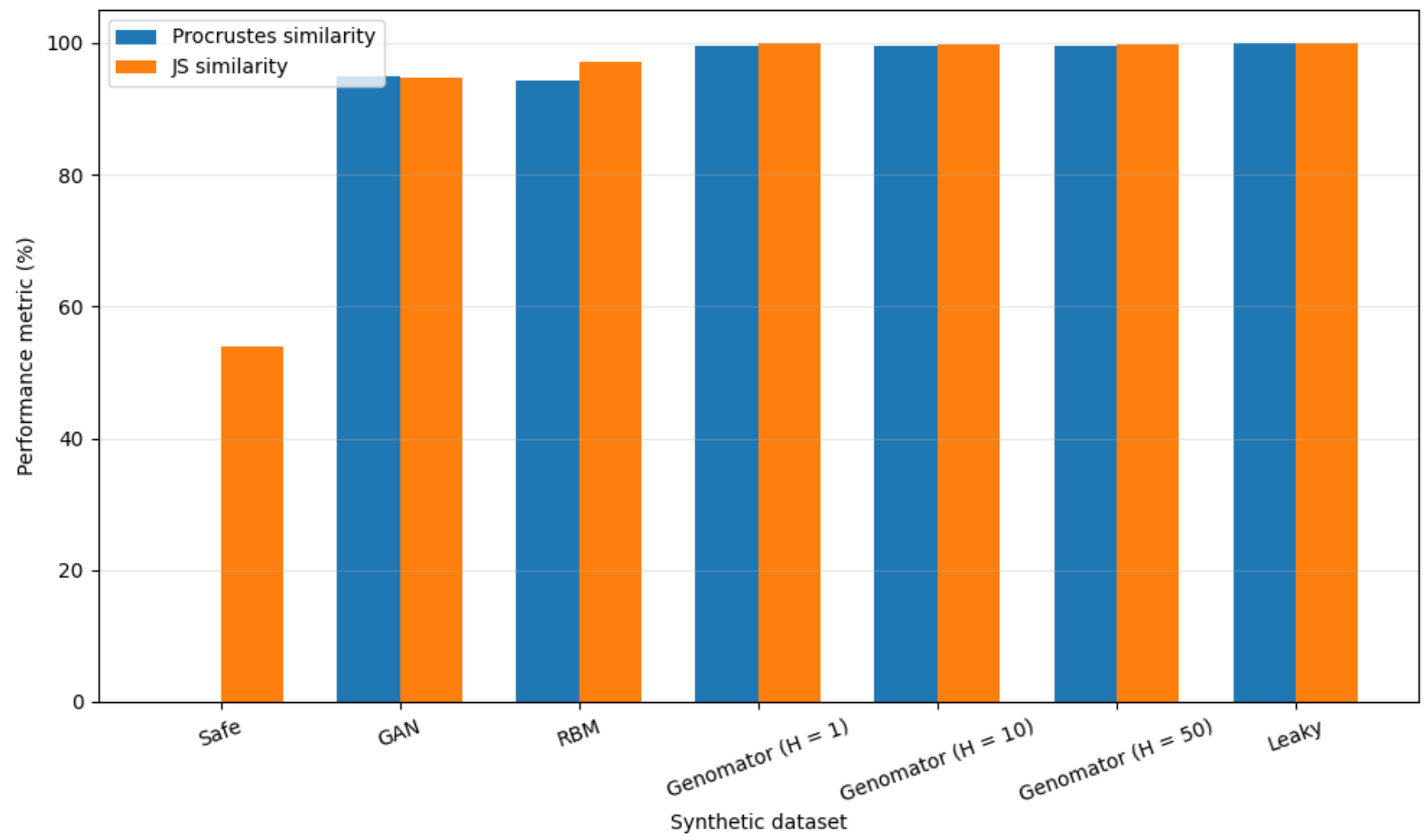

Figure 7: Performance of synthetic datasets on the chromosome 15 panel (10,000 SNPs) using genetic ancestry inference as the utility task. Utility is evaluated via Procrustes similarity between real and synthetic PCA embeddings and JS similarity of inferred ancestry proportions, comparing safe, generative (GAN, RBM, Genomator), and leaky baselines.

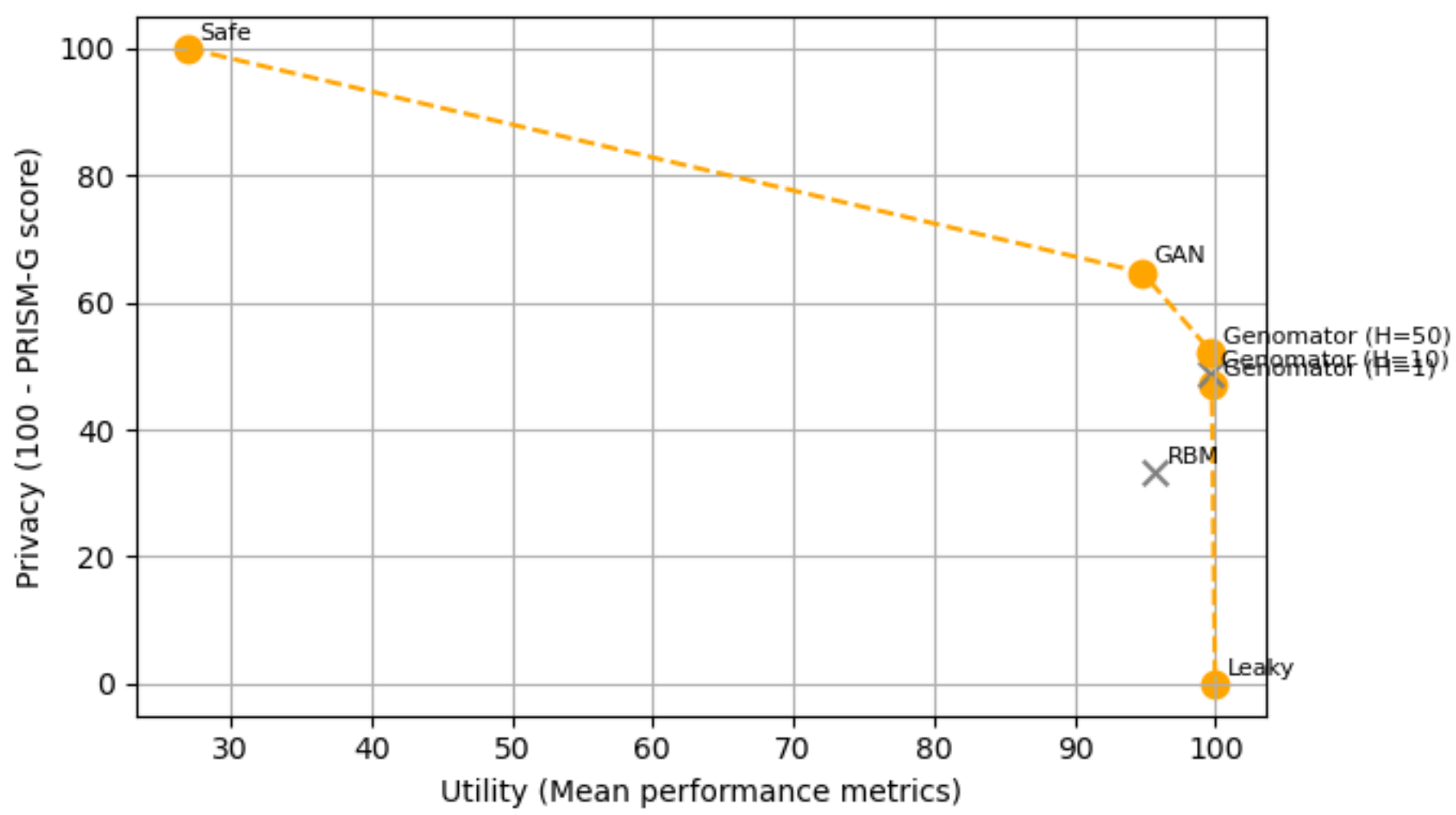

Figure 8: Privacy–utility trade-off for synthetic datasets generated from the chromosome 15 panel (10,000 SNPs). Utility is measured as the mean performance of the ancestry inference metrics, while privacy is quantified as PRISM-G score. The plot illustrates how generative models (GAN, RBM, Genomator) position between the safe baseline (high privacy, low utility) and the leaky baseline (high utility, minimal privacy), tracing the privacy–utility frontier.

References

[1] Nick Patterson, Alkes L. Price, and David Reich. "Population Structure and Eigenanalysis". en. In: *PLoS Genetics* 2.12 (2006), e190. ISSN: 1553-7390, 1553-7404. DOI: 10.1371/journal.pgen.0020190. URL: https://dx.plos.org/10.1371/journal.pgen.0020190 (visited on 09/24/2025).

[2] Andrew Yale et al. "Privacy Preserving Synthetic Health Data". en. In: ().

[3] Jérôme Goudet, Tomas Kay, and Bruce S. Weir. "How to estimate kinship". en. In: *Molecular Ecology* 27.20 (Oct. 2018), pp. 4121–4135. ISSN: 0962-1083, 1365-294X. DOI: 10.1111/mec.14833. URL: https://onlinelibrary.wiley.com/doi/10.1111/mec.14833 (visited on 10/09/2025).

[4] P.M. VanRaden. "Efficient Methods to Compute Genomic Predictions". en. In: *Journal of Dairy Science* 91.11 (Nov. 2008), pp. 4414–4423. ISSN: 00220302. DOI: 10.3168/jds.2007-0980. URL: https://linkinghub.elsevier.com/retrieve/pii/S0022030208709901 (visited on 12/02/2025).

[5] Ani Manichaikul et al. "Robust relationship inference in genome-wide association studies". en. In: *Bioinformatics* 26.22 (Nov. 2010), pp. 2867–2873. ISSN: 1367-4811, 1367-4803. DOI: 10.1093/bioinformatics/btq559. URL: https://academic.oup.com/bioinformatics/article/26/22/2867/228512 (visited on 09/24/2025).

[6] Fabio Oldoni, Kenneth K. Kidd, and Daniele Podini. "Microhaplotypes in forensic genetics". en. In: *Forensic Science International: Genetics* 38 (Jan. 2019), pp. 54–69. ISSN: 18724973. DOI: 10.1016/j.fsigen.2018.09.009. URL: https://linkinghub.elsevier.com/retrieve/pii/S1872497318303910 (visited on 10/09/2025).

[7] Daniel N. Seidman et al. "Rapid, Phase-free Detection of Long Identity-by-Descent Segments Enables Effective Relationship Classification". en. In: *The American Journal of Human Genetics* 106.4 (Apr. 2020), pp. 453–466. ISSN: 00029297. DOI: 10.1016/j.ajhg.2020.02.012. URL: https://linkinghub.elsevier.com/retrieve/pii/S0002929720300549 (visited on 10/09/2025).

[8] Bo Eskerod Madsen and Sharon R. Browning. "A Groupwise Association Test for Rare Mutations Using a Weighted Sum Statistic". en. In: *PLoS Genetics* 5.2 (Feb. 2009). Ed. by Nicholas J. Schork, e1000384. ISSN: 1553-7404. DOI: 10.1371/journal.pgen.1000384. URL: https://dx.plos.org/10.1371/journal.pgen.1000384 (visited on 09/24/2025).

[9] Suyash S. Shringarpure and Carlos D. Bustamante. "Privacy Risks from Genomic Data-Sharing Beacons". en. In: *The American Journal of Human Genetics* 97.5 (Nov. 2015), pp. 631–646. ISSN: 00029297. DOI: 10.1016/j.ajhg.2015.09.010. URL: https://linkinghub.elsevier.com/retrieve/pii/S0002929715003742 (visited on 09/24/2025).

[10] Burak Yelmen et al. "Creating artificial human genomes using generative neural networks". en. In: *PLOS Genetics* 17.2 (Feb. 2021). Ed. by Sara Mathieson, e1009303. ISSN: 1553-7404. DOI: 10.1371/journal.pgen.1009303. URL: https://dx.plos.org/10.1371/journal.pgen.1009303 (visited on 09/24/2025).

[11] Mark A Burgess et al. "Privacy-hardened and hallucination-resistant synthetic data generation with logic-solvers". en. In: *Bioinformatics* 41.12 (Dec. 2025). Ed. by Inanc Birol, btaf600. ISSN: 1367-4811. DOI: 10.1093/bioinformatics/btaf600. URL: https://academic.oup.com/bioinformatics/article/doi/10.1093/bioinformatics/btaf600/8314204 (visited on 01/09/2026).

[12] The 1000 Genomes Project Consortium et al. "A global reference for human genetic variation". en. In: *Nature* 526.7571 (Oct. 2015), pp. 68–74. ISSN: 0028-0836, 1476-4687. DOI: 10.1038/nature15393. URL: https://www.nature.com/articles/nature15393 (visited on 12/02/2025).
